# Source-based morphometry analyses yield associations between large-scale structural brain networks and psychopathic traits in incarcerated adolescents

**DOI:** 10.3389/fpsyg.2026.1884761

**Published:** 2026-09-29

**Authors:** J. Michael Maurer, Corey H. Allen, David D. Stephenson, Keith A. Harenski, Bethany G. Edwards, Nathaniel E. Anderson, Carla L. Harenski, Vince D. Calhoun, Michael F. Caldwell, Gregory J. Van Rybroek, Kent A. Kiehl

**Affiliations:** 1 The Mind Research Network (MRN), Albuquerque, NM, United States; 2 Tri-Institutional Center for Translational Research in Neuroimaging and Data Science (TReNDS), Georgia Institute of Technology, and Emory University, Georgia State University, Atlanta, GA, United States; 3 Mendota Mental Health Institute, Madison, WI, United States; 4 Department of Psychology, University of Wisconsin-Madison, Madison, WI, United States; 5 University of Wisconsin Law School, Madison, WI, United States; 6 Department of Psychology, University of New Mexico, Albuquerque, NM, United States

**Keywords:** juvenile delinquency, multivariate analyses, psychopathic traits, source-based morphometry, structural MRI

## Abstract

Studies commonly report that youth with elevated psychopathic traits exhibit deficits within limbic and paralimbic regions, including reduced gray matter volume (GMV) in the amygdala, orbitofrontal cortex (OFC), and temporal pole. However, studies have also reported additional structural abnormalities in regions extending beyond limbic and paralimbic regions among youth with elevated psychopathic traits, including the middle frontal gyrus (MFG), inferior frontal gyrus (IFG), precuneus, inferior parietal lobule (IPL), and fusiform gyrus. This suggests youth with elevated psychopathic traits may be associated with deficits within large-scale structural networks. The current study investigated whether youth with elevated psychopathic traits were associated with such deficits through the use of source-based morphometry (SBM), a multivariate analysis of structural magnetic resonance imaging data. SBM uses Independent Component Analysis (ICA) to identify distinct sets of brain regions that form different large-scale structural networks. We investigated whether youth psychopathy scores, assessed via the Hare Psychopathy Checklist: Youth Version (PCL:YV), were associated with SBM loading coefficients among *n* = 413 incarcerated boys. Compared to participants scoring low on the PCL:YV, participants scoring high on the PCL:YV contributed less (i.e., exhibited reduced GMV) to large-scale structural networks containing limbic and paralimbic regions (e.g., the amygdala, insula, OFC, temporal pole, and posterior cingulate) and additional regions (e.g., the IFG, precuneus, IPL, and fusiform gyrus). Through the use of multivariate SBM analyses, the current study helps further improve our understanding of structural abnormalities among youth with elevated psychopathic traits.

## Introduction

Youth with elevated psychopathic traits are characterized by interpersonal dysfunction (e.g., grandiose sense of self-worth, pathological lying, and manipulation for personal gain) and pronounced affective deficits (e.g., shallow affect, and a lack of empathy, guilt, and remorse) (Forth et al., 2003). Together, these interpersonal and affective traits are commonly referred to as callous-unemotional (CU) traits (Frick, 2009). Beyond CU traits, youth with elevated psychopathic traits are also characterized by a disinhibited, impulsive, and irresponsible lifestyle (Maurer et al., 2021, 2024a) and prior history of significant antisocial behavior (e.g., engaging in early and serious problematic criminal behavior) (Forth et al., 2003). Compared to youth meeting criteria for externalizing disorders (e.g., conduct disorder or oppositional defiant disorder), youth with elevated psychopathic traits are believed to represent life-course persistent antisocial offenders, continuing to engage in high rates of delinquent behavior throughout their lifetime (Lussier et al., 2022; Piquero et al., 2012). Psychopathic traits assessed during adolescence are predictive of future adult criminal activity (Colins et al., 2015), recidivism (Allen et al., 2024a,b), and other deleterious outcomes, including hazardous substance use (Colins et al., 2015) and premature mortality (Maurer et al., 2024b). Given the severity of these consequences, it is imperative that researchers identify youth with elevated psychopathic traits early in development, as they may particularly benefit from specialized treatment intervention approaches. Indeed, the prevailing view is that intervention efforts targeted toward youth with elevated psychopathic traits have a better chance of altering life-course persistent antisocial behavior if such interventions are started early in life (Caldwell, 2011; Caldwell et al., 2006, 2007).

Researchers have identified that youth with elevated psychopathic traits exhibit deficits across multiple domains, including processing affective faces and words (see Díaz-Vazquez et al., 2024 for a review), sharing the emotional experiences of other individuals (i.e., deficits relating to affective empathy, rather than cognitive empathy; Dadds et al., 2009; Marsh et al., 2013), moral processing deficits (Harenski et al., 2014), difficulty regulating emotions (Heinzen et al., 2011), rewards processing deficits (Morgan et al., 2014; Reidy et al., 2017), attention deficits (Vitale et al., 2005), error-related processing deficits (Maurer et al., 2016), and failure to learn from punishment cues (Byrd et al., 2014; Salekin, 2017). Existing neurobiological theories suggest that limbic and paralimbic dysfunction contributes to these deficits among youth with elevated psychopathic traits. For example, Blair (Blair, 2003, 2007, 2013) suggested that abnormalities within the amygdala and ventromedial prefrontal cortex (vmPFC) contribute to both affective and empathy deficits among individuals with elevated psychopathic traits. Kiehl’s paralimbic hypothesis (Kiehl, 2006) further suggested that in addition to amygdala and vmPFC abnormalities, individuals with elevated psychopathic traits also exhibit deficits in additional limbic and surrounding paralimbic regions, including the orbitofrontal cortex (OFC), insula, parahippocampal gyrus, temporal pole, anterior cingulate cortex (ACC), and posterior cingulate cortex (PCC). Supporting both neurobiological theories, limbic and paralimbic regions contribute to multiple cognitive processes implicated in youth with elevated psychopathic traits, including affective processing (Deen et al., 2024; Maddock et al., 2003; Rolls and Grabenhorst, 2008; Todorov, 2012), empathy (Fallon et al., 2020; Fan et al., 2011), emotion regulation (Berboth and Morawetz, 2021; Frank et al., 2014; Golkar et al., 2012), rewards processing (Murray, 2007; Rolls, 2000), and learning from punishments (Murty et al., 2016; Orsini et al., 2015).

Studies incorporating structural magnetic resonance imaging (sMRI) have further supported these neurobiological theories, identifying deficits within limbic and paralimbic regions among youth with elevated psychopathic traits. For example, youth scoring high on measures of psychopathy exhibit reduced gray matter volume/concentration (GMV/GMC, respectively), surface area (SA), and cortical thickness (CT) in limbic and paralimbic regions, including the amygdala (Aghajani et al., 2017; Caldwell et al., 2019; Cohn et al., 2016; Cope et al., 2014; Ermer et al., 2013; Gao et al., 2024; Waller et al., 2020; Walters and Kiehl, 2015), insula (Caldwell et al., 2019; Cohn et al., 2016; Fairchild et al., 2013; Gao et al., 2024; Waller et al., 2020), parahippocampal gyrus (Bashford-Largo et al., 2025; Cope et al., 2014; Ermer et al., 2013; Steele et al., 2017), OFC (Bashford-Largo et al., 2025; Bolhuis et al., 2019; Caldwell et al., 2019; Cope et al., 2014; Ermer et al., 2013; Gao et al., 2024), temporal pole (Bashford-Largo et al., 2025; Bolhuis et al., 2019; Caldwell et al., 2019; Cope et al., 2014; Ermer et al., 2013; Gao et al., 2024; Steele et al., 2017), ACC (Gao et al., 2024; Sebastian et al., 2016), and PCC (Caldwell et al., 2019; Ermer et al., 2013).

However, additional regions extending beyond limbic and paralimbic regions contribute to cognitive processes implicated among youth with elevated psychopathic traits. For example, regions including the superior frontal gyrus (SFG), middle frontal gyrus (MFG), inferior frontal gyrus (IFG), fusiform gyrus, precuneus, precentral gyrus, and inferior parietal lobule (IPL) contribute to multiple cognitive processes, including processing emotionally salient faces (Fusar-Poli et al., 2009; Müller et al., 2018) and words (Brooks et al., 2016; Kuchinke et al., 2005), empathy capacity (Fallon et al., 2020; Xiong et al., 2019), emotion regulation (Kohn et al., 2014; Ochsner et al., 2012), moral processing (Boccia et al., 2017), reorienting attention (Japee et al., 2015; Li et al., 2020), and error processing (Iannaccone et al., 2015; Steele et al., 2014). Studies incorporating sMRI have similarly observed deficits within these regions among youth with elevated psychopathic traits, including reduced GMV/GMC, CT, and SA in the SFG (Bolhuis et al., 2019; Waller et al., 2020), MFG (Gao et al., 2024; Jiang et al., 2023; Sebastian et al., 2016), IPL (Ermer et al., 2013; Gao et al., 2024; Yang et al., 2015), precuneus (Caldwell et al., 2019; Gao et al., 2024), and fusiform gyrus (Bolhuis et al., 2019). These results suggest that youth with elevated psychopathic traits may not only be associated with deficits within circumscribed limbic and paralimbic regions. Instead, youth with elevated psychopathic traits may exhibit structural deficits within widespread brain regions, including both limbic and paralimbic regions (e.g., OFC, insula, amygdala, temporal pole, ACC, PCC, and parahippocampal gyrus) and additional regions, including frontal regions (e.g., SFG, IFG), temporal regions (e.g., fusiform gyrus), and parietal regions (e.g., precuneus and IPL). Beyond these individual regions of interest (ROIs), youth with elevated psychopathic traits may be associated with reduced structural coherence between these regions, exhibiting deficits within large-scale structural networks. However, no study to date has explored whether youth with elevated psychopathic traits are associated with reduced structural coherence between brain regions previously identified when incorporating univariate statistical analyses (i.e., voxel-based morphometry; VBM).

Source-based morphometry (SBM; Xu et al., 2009) is a multivariate approach ideally suited to investigate whether youth with elevated psychopathic traits are associated with deficits within large-scale structural networks. SBM incorporates the use of Independent Component Analysis (ICA) to identify distinct sets of brain regions that covary together, forming different large-scale structural networks. Both univariate (e.g., VBM) and multivariate approaches allow for the identification of brain regions which significantly relate to variables of interest or differ between groups; however, only multivariate approaches, such as SBM, allow for the identification of relationships between these brain regions (i.e., structural covariance or structural connectivity; Gupta et al., 2019). Univariate approaches, including VBM, identify structural deficits by analyzing each voxel independently. Univariate approaches are therefore effective at identifying local changes within specific ROIs, but are less effective at capturing distributed patterns of variation (Radua et al., 2014). In contrast, multivariate approaches like SBM are able to measure covariance among voxels across the entire brain, allowing for the opportunity to investigate structural connectivity between widespread and distant brain regions. The large-scale structural networks obtained via SBM correspond closely to resting-state networks obtained from functional MRI (fMRI) analyses (Segall et al., 2012). Structural networks obtained via SBM have improved our understanding of structural abnormalities across multiple conditions, including schizophrenia (Huang et al., 2025; Kaspárek et al., 2010; Xu et al., 2009), Parkinson’s Disease (Samantaray et al., 2024; Vacca et al., 2025), substance use (Anderson et al., 2025; Lacomba-Arnau et al., 2025), sadism (Harenski et al., 2025), and multiple sclerosis (Bergsland et al., 2018). Furthermore, structural networks obtained via SBM have been shown to be predictive of future antisocial outcomes, including recidivism (Kiehl et al., 2018). SBM may therefore identify large-scale structural networks implicated among youth with elevated psychopathic traits, which can be targeted using specialized treatment intervention approaches designed to help reduce life-course persistent antisocial offending among this at-risk group.

The current study aimed to improve our understanding regarding widespread structural deficits among incarcerated youth with elevated psychopathic traits using multivariate statistical analyses (i.e., SBM) of sMRI data. Psychopathic traits were assessed using the Hare Psychopathy Checklist: Youth Version (PCL:YV; Forth et al., 2003). We hypothesized that compared to participants scoring low on the PCL:YV, participants scoring high on the PCL:YV would contribute less (i.e., exhibit reduced GMV) to large-scale structural networks comprised of hypothesized limbic and surrounding paralimbic regions proposed in existing neurobiological theories (e.g., the amygdala, OFC, temporal pole, parahippocampal gyrus, insula, ACC, and PCC). We additionally hypothesized that these same large-scale structural networks would additionally contain regions extending beyond hypothesized limbic and paralimbic regions (e.g., the SFG, MFG, IFG, IPL, and precentral gyrus). Through the use of multivariate SBM analyses, we aimed to extend upon prior research, investigating whether youth with elevated psychopathic traits were associated with reduced structural coherence within large-scale structural networks comprised of limbic/paralimbic, frontal, parietal, and temporal brain regions.

## Materials and method

### Participants

Participants included *n* = 413 incarcerated boys recruited from a maximum-security juvenile correctional facility in New Mexico and the Mendota Juvenile Treatment Center (MJTC) in Wisconsin. Participants ranged from 13.82 to 20.76 years of age (*M* = 17.14 years, SD = 1.19 years). Participants self-identified as American Indian or Alaskan Native (*n* = 31), Black or African American (*n* = 108), Native Hawaiian or other Pacific Islander (*n* = 1), White (*n* = 203), more than one race (*n* = 31), Hispanic or Latino (*n* = 199), or not Hispanic or Latino (*n* = 210). Thirty-nine participants chose not to self-report their race and four participants chose not to self-disclose their ethnicity. Participants included in the current study overlap with prior studies from our research group, which incorporated univariate statistical analyses investigating the association between PCL:YV scores and GMV among *n* = 191 incarcerated boys (see Ermer et al., 2013; Steele et al., 2017; Walters and Kiehl, 2015).

Initial contact was made with study participants by research staff from the Mind Research Network and informed consent was obtained. All participants and their parents/guardian or resident advocate provided written informed consent. Most parents of MJTC participants could not be reached because they were unknown, their parental rights were terminated, and/or the participants were wards of the state. In these situations, the resident advocates acting in a limited guardianship role signed the written informed consent form. Participants received payment consistent with the hourly labor wage of the facility at which they were housed. All research protocols were approved by the Ethical and Independent Review Services, the Office for Human Research Protections, the University of New Mexico Human Research Protections Office, University of Wisconsin-Madison, and the New Mexico and Wisconsin Department of Corrections.

### Assessments

Youth psychopathic traits were assessed using the PCL:YV (Forth et al., 2003), a 20-item expert-rater assessment, consisting of a semi-structured interview and review of collateral information, including institutional file review. Each of the twenty items of the PCL:YV are scored based on the following rating scale: zero (does not apply to the individual), one (applies somewhat to the individual), or two (definitely applies to the individual). PCL:YV total scores can potentially range from zero to 40; the mean PCL:YV total score in the current sample was 25.10 (SD = 6.09, range: 2–38; α = 0.80). Factor analysis of the PCL:YV reveals a reliable two-factor and four-facet solution, with Factor 1 consisting of interpersonal (i.e., Facet 1) and affective (i.e., Facet 2) psychopathic traits and Factor 2 including lifestyle/behavioral (i.e., Facet 3) and antisocial/developmental (i.e., Facet 4) psychopathic traits (Forth et al., 2003; Neumann et al., 2006). Our primary analyses investigated the association between PCL:YV total scores and SBM loading coefficients. We performed additional analyses, described in further detail in our Supplementary material, investigating the association between PCL:YV factor and facet scores and SBM loading coefficients (see Supplementary Tables 2, 3).

In addition to youth psychopathic traits, we assessed severity of alcohol and illicit substance use using a modified version of the Addiction Severity Index (ASI; McLellan et al., 1992). Youth with elevated psychopathic traits are routinely characterized by comorbid substance use (see Sakki et al., 2023 for a review); additionally, individuals with more severe patterns of substance use exhibit structural abnormalities in limbic and paralimbic regions (Pando-Naude et al., 2021; Yan et al., 2023). Substance use severity was therefore included as a covariate measure in multiple regression analyses, to ensure that significant effects were specifically associated with youth psychopathic traits, rather than comorbid substance use. With the modified ASI, years of use were summed across all substances that the participant reported using regularly (i.e., three or more times per week for a minimum period of 1 month). Substances assessed included alcohol, cannabis, heroin, cocaine, methamphetamine or other amphetamines, hallucinogens, inhalants, methadone, other opiates/analgesics (e.g., OxyContin, Percocet, etc.), and other substances (e.g., ecstasy, etc.). Years of regular substance use were divided by the participant’s age to control for opportunity to use, multiplied by 100, and a square root transformation was applied to correct for skewness. Substance use severity scores ranged from 0 to 18.64 (*M* = 4.93, SD = 3.24). We have previously incorporated this operational definition of substance use severity as a covariate measure in prior neuroimaging studies (Cope et al., 2014; Ermer et al., 2013). See Table 1 for descriptive statistics for all variables included in analyses and Supplementary Table 1 for all correlations between study variables.

**TABLE 1:** Descriptive statistics for PCL:YV scores and covariate measures.

Variable	M	SD	Range	Skewness	Kurtosis
PCL:YV total	25.10	6.09	2–38	−0.34	−0.04
PCL:YV Factor 1	7.82	3.37	0–16	0.10	−0.66
PCL:YV Factor 2	14.93	2.99	1–20	−0.99	1.91
PCL:YV Facet 1	2.80	2.09	0–8	0.49	−0.75
PCL:YV Facet 2	5.02	1.86	0–8	−0.36	−0.59
PCL:YV Facet 3	6.66	1.93	0–10	−0.50	−0.10
PCL:YV Facet 4	8.27	1.63	0–10	−1.28	2.46
Substance use severity	4.93	3.24	0–18.64	0.24	−0.03
Age at MRI scan	17.14	1.19	13.82–20.76	−0.21	−0.37
Brain volume	1229.02	100.71	967.97–1555.70	0.22	0.16

PCL:YV refers to scores from the Hare Psychopathy Checklist: Youth Version (Forth et al., 2003); substance use severity scores were obtained using a modified version of the Addiction Severity Index (ASI; McLellan et al., 1992); brain volume refers to the sum of each participant’s gray and white matter volume.

### MRI acquisition

High-resolution T1-weighted structural MRI scans were acquired using the Mind Research Network’s 1.5T Avanto mobile MRI scanner, using a multi-echo pulse sequence provided by the Massachusetts General Hospital Radiology Department (TE’s = 1.64 ms, 3.50 ms, 5.36 ms, 7.22 ms, TR = 2530 ms, inversion time = 1100 ms, flip angle = 7°, slice thickness = 1.3 mm, matrix size = 256 × 256) yielding 128 sagittal slices with an in-plane resolution of 1.0 mm × 1.0 mm. Data were preprocessed and analyzed using Statistical Parametric Mapping software (SPM12)^1^. T1-images were automatically reoriented to the anterior-posterior commissure alignment using the auto_acpc_reorient algorithm^2^ and visually inspected to ensure proper alignment.

Images were then analyzed using the Unified Segmentation approach implemented in SPM12 (Ashburner and Friston, 2005), using the default tissue probability map (TPM.nii). This tissue probability map (TPM) contains six tissue partitions (gray, white, cerebrospinal fluid [CSF], bone, soft tissue, and other). Unified Segmentation allows for image registration based on Gaussian mixture modeling, tissue classification with warped prior probability maps, and bias correction to be combined in the same generative model. During spatial normalization, data were resampled to 2 × 2 × 2 mm. Subsequent segmentation partitioned the images into gray matter, white matter, and CSF, which were then modulated with Jacobian determinants to preserve total volume (Ashburner and Friston, 2000, 2005). Voxel-wise parameters can therefore be interpreted as GMV. Visual inspection was conducted before and after segmentation and normalization to ensure the quality of each image. Images were smoothed with a 10 mm full width at half-maximum (FWHM) Gaussian kernel to reduce false positive rates (Silver et al., 2011). The original SBM source paper (Xu et al., 2009) incorporated similar smoothing levels (12 mm FWHM) and subsequent SBM publications have similarly incorporated 10 mm FWHM smoothing (Harenski et al., 2020; Kiehl et al., 2018; Kolla et al., 2021; Saha et al., 2023). Voxels with a gray matter value <0.15 were excluded to remove possible partial volume effects between gray and white matter. All structural T1 images were further assessed using the image quality metrics (IQMs) produced by the MRIQC toolbox (Esteban et al., 2017), including the signal-to-noise ratio (SNR), the coefficient of joint variation between gray and white matter (CJV), and the entropy focus condition (EFC). None of the *n* = 413 participants included in the current study were characterized as extreme outliers according to IQMs (i.e., 1.5 inter-quartile range [IQR] below the first quartile or above the third quartile), consistent with prior structural MRI studies (e.g., Baranger et al., 2021; Monereo-Sánchez et al., 2021).

### Source-based morphometry

SBM is a data-driven, model-free, multivariate analysis of sMRI data which incorporates ICA to identify intrinsic structural networks of covarying gray matter (Xu et al., 2009). Following preprocessing, the Group ICA of fMRI Toolbox (GIFT) software (Group ICAT v4.0.5.18)^3^ was used to extract 30 maximally independent components (Xu et al., 2009). The original SBM source paper (Xu et al., 2009) suggests that roughly 30 SBM components are needed for a reliable ICA solution relating to sMRI data. Subsequently published SBM papers have also chosen 30 overall SBM components in analyses performed (Anderson et al., 2019; DeRamus et al., 2020; Kiehl et al., 2018; Maurer et al., 2025). Group ICA was run considering all *n* = 413 study participants to obtain a common set of sources with the neural network algorithm (InfoMax) that attempts to maximize the mutual information of the network outputs (McKeown et al., 1998). The InfoMax algorithm produces a set of maximally independent components of covarying voxels and parameter values for each participant, indicating how well their data contributes to the ICA solution. Stronger or weaker parameter values (i.e., loading coefficients) scale proportionately with individual gray matter values in regions represented in a given SBM component. These loading coefficients can then be related to individual variables of interest (e.g., PCL:YV scores, covariate measures, etc.). The statistical reliability of the source decomposition was tested using the ICASSO toolbox (Himberg and Hyvärinen, 2003) by running InfoMax ten times (Wei et al., 2022), with different initial conditions using both the RandInt and bootstrapped options. ICASSO estimation provided very good reliability of the InfoMax algorithm, as indexed by the mean stability index across the 30 SBM components (0.96 ± 0.03). Individual source maps were then converted to Z-scores before running subsequent statistics. Source matrix was used for visualization, scaling each map to unit standard deviation (SBM Z-map) and thresholded using a Z-score threshold of ≥1.0.

### Data analysis

Our current study incorporated both planned comparisons and exploratory analyses. Regarding planned comparisons, we first investigated whether PCL:YV total scores were significantly correlated with loading coefficients obtained from SBM components containing hypothesized limbic and paralimbic regions (e.g., OFC, amygdala, insula, parahippocampal gyrus, ACC, PCC, and temporal pole). We then performed subsequent multiple regression analyses, investigating whether PCL:YV total scores remained associated with SBM loading coefficients when including additional covariate measures as simultaneous predictor variables. Covariates entered in multiple regression analyses included substance use severity scores, participant’s age at MRI data collection, and total brain volume (i.e., the sum of each participant’s total gray and white matter volume). We also performed exploratory correlation analyses, investigating whether PCL:YV total scores were significantly correlated with loading coefficients obtained from SBM components which did not contain hypothesized limbic and paralimbic regions. Prior research (e.g., Barnett et al., 2022; Streiner and Norman, 2011) argues multiple comparison correction is not needed for planned comparisons; however, we additionally report which of our planned comparisons survived multiple comparison correction with footnotes in our Results section. With our exploratory analyses, we incorporated multiple comparison corrections, utilizing a modified Bonferroni multiple comparison correction (more details included below). We additionally performed correlation and multiple regression analyses using PCL:YV factor and facet scores, which are described in further detail in our Supplementary material.

## Results

### SBM results

SBM using ICA generated 30 independent components. Eight of these 30 SBM components were determined to reflect artifacts based on the criteria established by the original SBM source paper (Xu et al., 2009), including SBM components containing sharp edges near the boundary of the brain or appearing in regions that do not contain gray matter (e.g., white matter or ventricles). Analyses were therefore conducted using the remaining 22 non-artifactual SBM components.

### Correlation analyses

First, we investigated whether PCL:YV total scores were significantly correlated with loading coefficients obtained from non-artifactual SBM components containing limbic and paralimbic regions, consistent with existing neurobiological theories (Blair, 2003, 2007, 2013; Kiehl, 2006). Out of the 22 non-artifactual SBM components, we identified four SBM components containing hypothesized limbic and paralimbic regions including the amygdala, OFC, insula, parahippocampal gyrus, temporal pole, ACC, and PCC: SBM Components 6, 12, 28, and 29. Each of these four SBM components additionally contained brain regions extending beyond limbic and paralimbic regions, including frontal regions (e.g., the SFG, IFG, MFG, and precentral gyrus), parietal regions (e.g., precuneus, supramarginal gyrus, and IPL), temporal/occipital regions (e.g., fusiform gyrus), and subregions of the cerebellum (e.g., uvula and vermis). While some regions were only included within single SBM components (e.g., the OFC and amygdala were only included in SBM Component 12 and the PCC was only included in SBM Component 29), other regions were shared across each of the four SBM components, including the temporal pole, supramarginal gyrus, and precentral gyrus. Additionally, other regions were included within at least three of the four SBM components, including the parahippocampal gyrus, SFG, MFG, IFG, precuneus, fusiform gyrus, and the IPL. See Figures 1–4 for a visual representation of each of these four SBM components and Supplementary Tables 4–7 for a full list of brain regions included within these four SBM components. Additionally, we further validated the brain regions included within each of these four SBM components by investigating the total number of voxels included within Automated Anatomical Labeling Atlas 3 (AAL3; Rolls et al., 2020) ROIs using absolute value Z-score thresholds ranging from 1.0 to 4.0 (see Supplementary Figures 1–16). Despite these four SBM components containing similar brain regions, each of these SBM components correspond to different large-scale structural networks. SBM Component 6 largely corresponded to a medial frontal/anterior cingulate structural pattern outlined in the color red and a fronto-temporo-parietal structural pattern outlined in the color blue. SBM Component 12 represented an occipital-cerebellar structural pattern outlined in the color red and a fronto-temporo-parietal structural pattern extending to the cerebellum outlined in the color blue. SBM Component 28 largely corresponded to a frontal structural pattern outlined in the color red and a temporo-parietal-occipital structural pattern outlined in the color blue, whereas SBM Component 29 represented a fronto-occipital structural pattern outlined in the color red and a fronto-temporo-parietal-occipital structural pattern outlined in the color blue.

**FIGURE 1:**
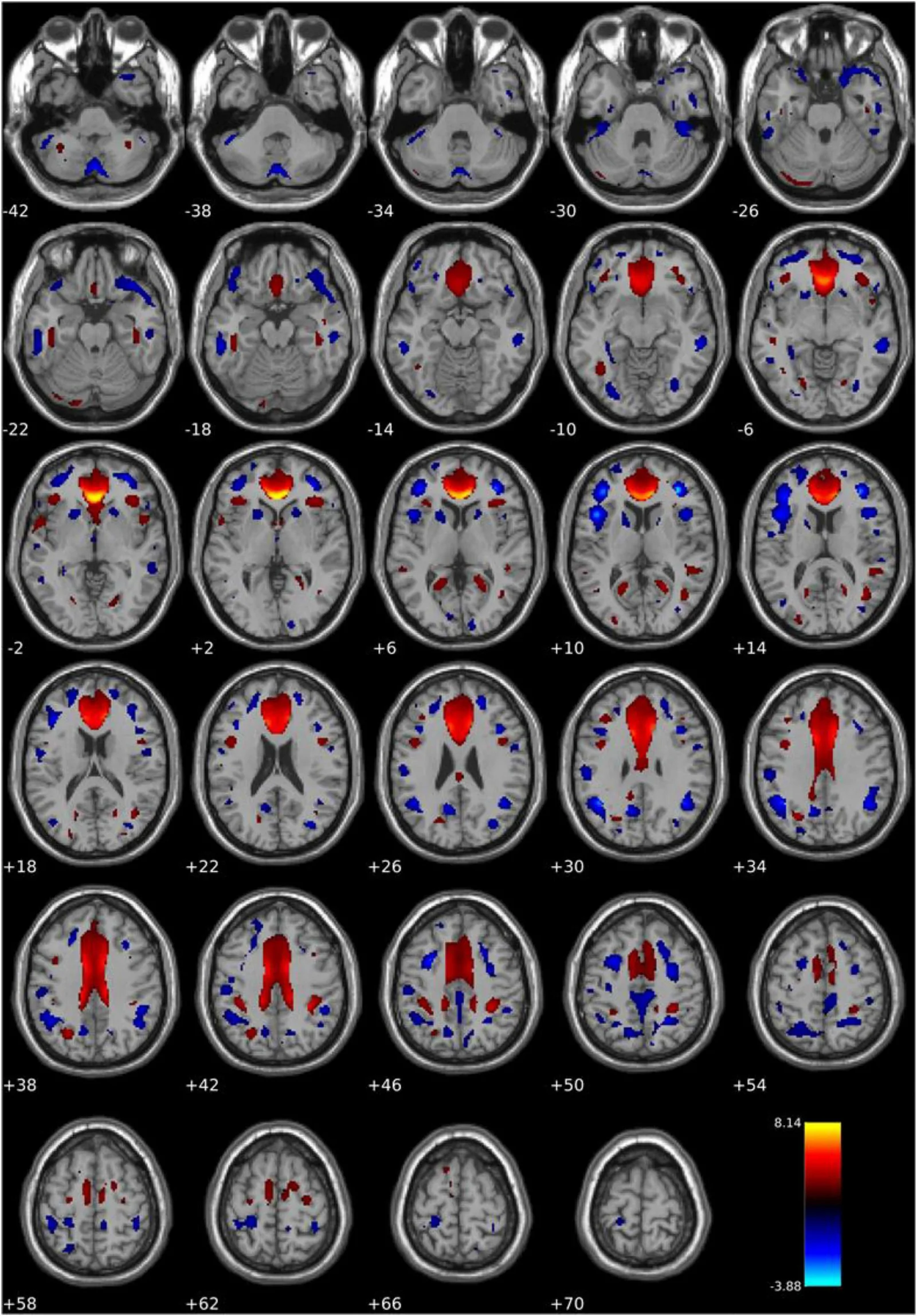
Visual representation of brain regions included within SBM Component 6. Autoslice image showing the brain regions included in SBM Component 6. Results are shown using a Z-score threshold of ≥1.0. SBM Component 6 largely corresponds to a medial frontal/anterior cingulate structural pattern (regions in the color red) expressed more among participants scoring high on the PCL:YV and a fronto-temporo-parietal structural pattern (regions in the color blue) expressed less among participants scoring high on the PCL:YV.

**FIGURE 2:**
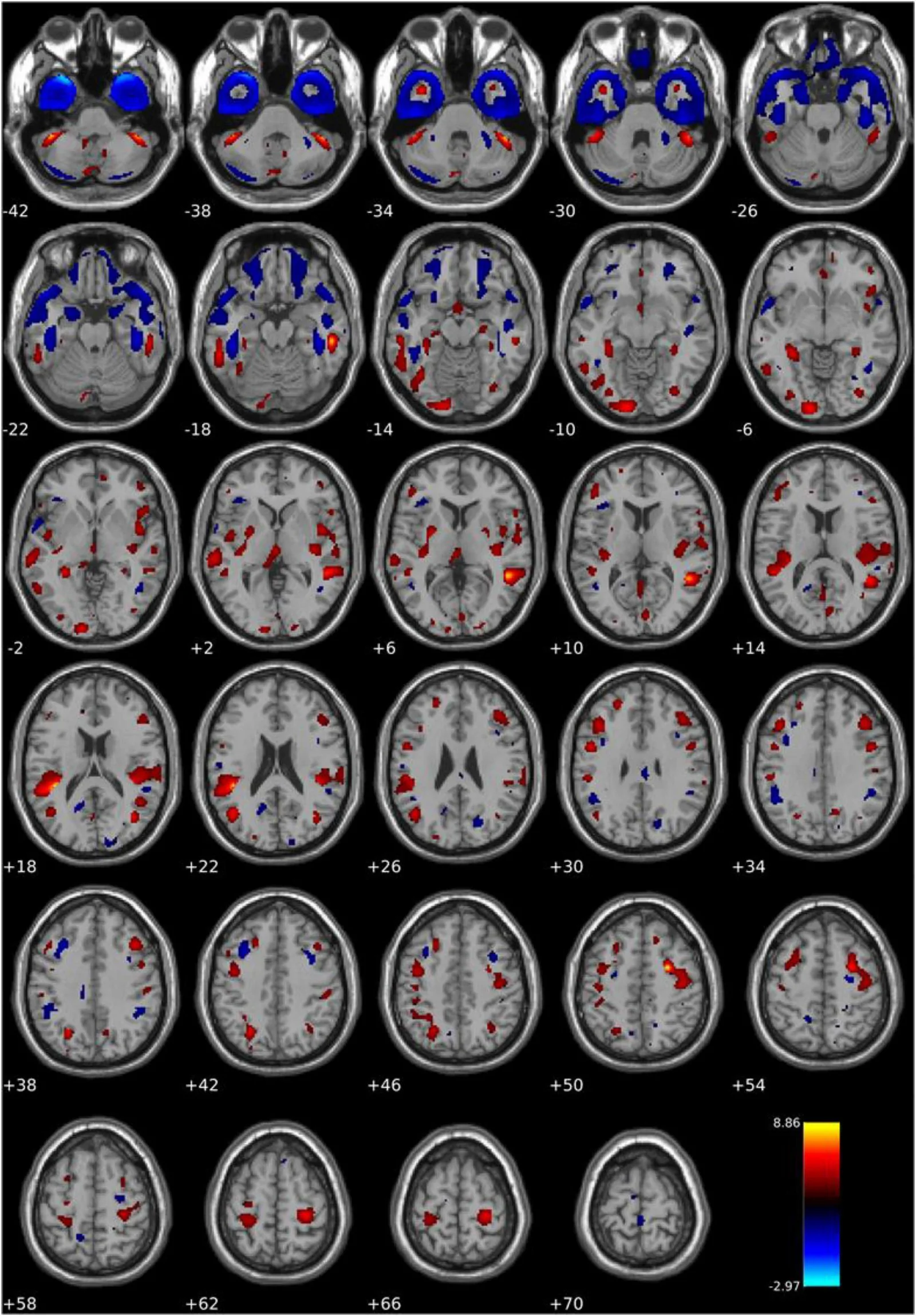
Visual representation of brain regions included within SBM Component 12. Autoslice image showing the brain regions included in SBM Component 12. Results are shown using a Z-score threshold of ≥1.0. SBM Component 12 largely corresponds to an occipital-cerebellar structural pattern (regions in the color red) expressed more among participants scoring high on the PCL:YV and a fronto-temporo-parietal structural pattern (regions in the color blue) expressed less among participants scoring high on the PCL:YV.

**FIGURE 3:**
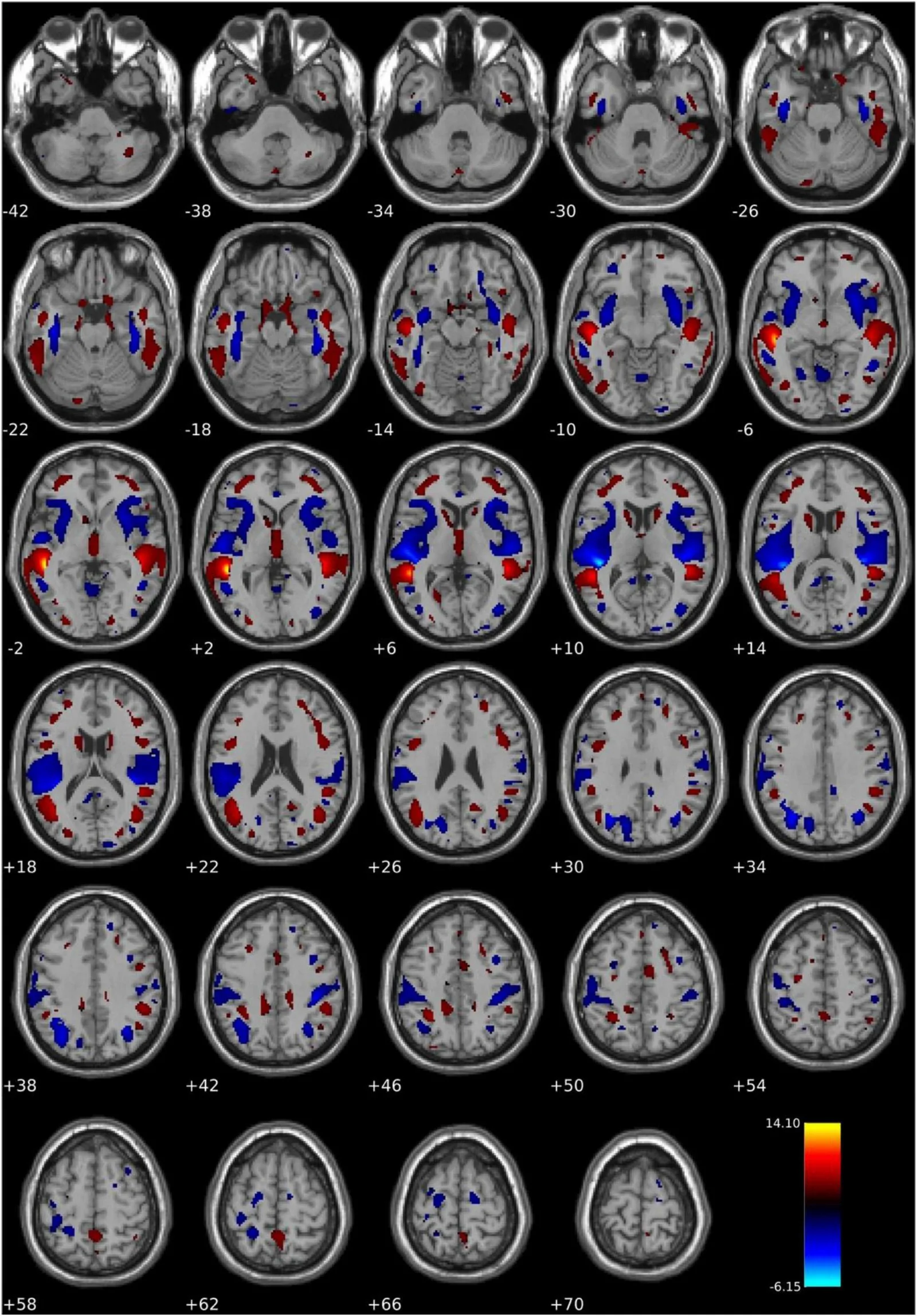
Visual representation of brain regions included within SBM Component 28. Autoslice image showing the brain regions included in SBM Component 28. Results are shown using a Z-score threshold of ≥1.0. SBM Component 28 largely corresponds to a frontal structural pattern (regions in the color red) expressed more among participants scoring high on the PCL:YV and a temporo-parietal-occipital structural pattern (regions in the color blue) expressed less among participants scoring high on the PCL:YV.

**FIGURE 4:**
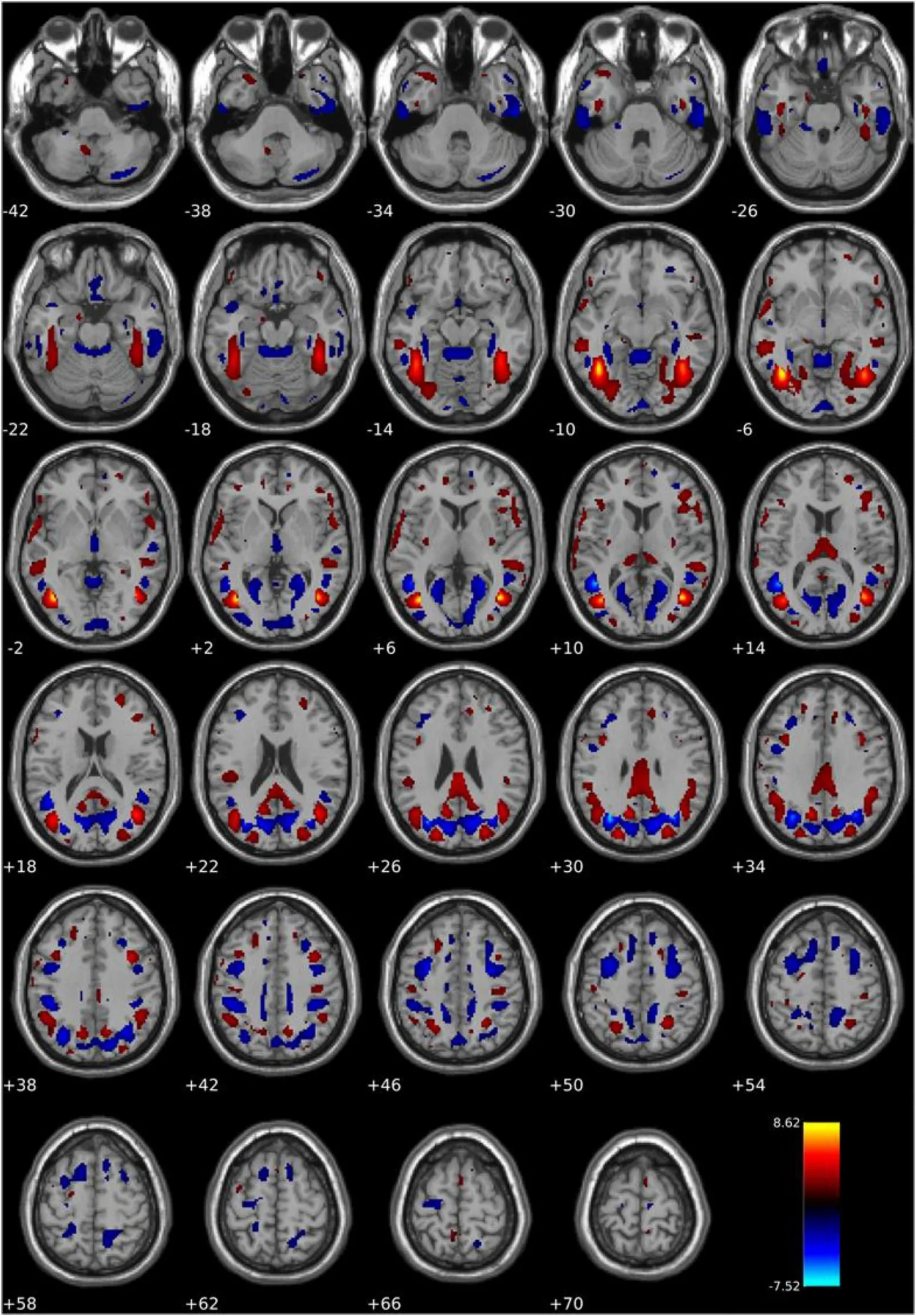
Visual representation of brain regions included within SBM Component 29. Autoslice image showing the brain regions included in SBM Component 29. Results are shown using a Z-score threshold of ≥1.0. SBM Component 29 largely corresponds to a fronto-occipital structural pattern (regions in the color red) expressed more among participants scoring high on the PCL:YV and a fronto-temporo-parietal-occipital structural pattern (regions in the color blue) expressed less among participants scoring high on the PCL:YV.

We observed that PCL:YV total scores were positively correlated with loading coefficients obtained from SBM Component 6 (*r* = 0.14, *p* = 0.006), SBM Component 12 (*r* = 0.13, *p* = 0.009), SBM Component 28 (*r* = 0.14, *p* = 0.003), and SBM Component 29 (*r* = 0.11, *p* = 0.022)^4^. Regarding the directionality of SBM effects, our results suggest that participants scoring high on the PCL:YV contributed *more* to regions outlined in red and *less* to regions outlined in blue within these four SBM components compared to participants scoring low on the PCL:YV. These results suggest that participants scoring high on the PCL:YV exhibited increased GMV within regions such as the ACC, cingulate gyrus, and medial prefrontal cortex (presented in the color red) and reduced GMV in regions such as the amygdala, OFC, temporal pole, insula, parahippocampal gyrus, temporal pole, PCC, SFG, IFG, MFG, fusiform gyrus, precuneus, supramarginal gyrus, precentral gyrus, IPL and cerebellar subregions (presented in the color blue) compared to participants scoring low on the PCL:YV.

Additionally, we investigated whether PCL:YV total scores were significantly correlated with the remaining 18 non-artifactual SBM components which did not include hypothesized limbic and paralimbic regions. Given the exploratory nature of these analyses, we implemented a modified Bonferroni multiple comparison correction (i.e., 0.05/18 or *p* < 0.003), as suggested by others (Barnett et al., 2022; Streiner and Norman, 2011). PCL:YV total scores were not significantly correlated with loading coefficients obtained from these remaining 18 non-artifactual SBM components (i.e., *p*’s > 0.003).

### Multiple regression analyses

We further tested the robustness of the association between PCL:YV total scores and SBM loading coefficients with multiple regression analyses. Here, loading coefficients from SBM Component 6 (Regression #1), SBM Component 12 (Regression #2), SBM Component 28 (Regression #3), and SBM Component 29 (Regression #4) were entered as separate dependent measures. Simultaneous predictor variables included PCL:YV total scores, substance use severity scores, participant’s age at MRI data collection, and total brain volume. Consistent with correlation analyses, PCL:YV total scores remained associated with loading coefficients obtained from SBM Component 6 (β = 0.13, *p* = 0.011), SBM Component 12 (β = 0.13, *p* = 0.012), SBM Component 28 (β = 0.16, *p* = 0.002), and SBM Component 29 (β = 0.12, *p* = 0.019), even when including additional covariate measures in multiple regression analyses (see Table 2)^5^.

**TABLE 2:** Multiple regression analyses.

Variable	B	SE(B)	β	*t*	*P*-value
Regression #1:					
SBM Component 6					
PCL:YV total	0.02	0.01	0.13	2.55	0.011**
Substance use severity	−0.01	0.02	−0.02	−0.40	0.690
Age at MRI scan	−0.04	0.04	−0.04	−0.84	0.401
Brain volume	0.00	0.00	0.05	1.04	0.299
Regression #2:					
SBM Component 12					
PCL:YV total	0.02	0.01	0.13	2.52	0.012**
Substance use severity	−0.02	0.02	−0.07	−1.41	0.160
Age at MRI scan	−0.06	0.04	−0.08	−1.51	0.133
Brain volume	0.00	0.00	0.15	3.12	0.002**
Regression #3:					
SBM component 28					
PCL:YV total	0.03	0.01	0.16	3.11	0.002**
Substance use severity	−0.03	0.02	−0.11	−2.18	0.030*
Age at MRI scan	−0.02	0.04	−0.02	−0.41	0.679
Brain volume	−0.00	0.00	−0.14	−2.79	0.005**
Regression #4:					
SBM Component 29					
PCL:YV total	0.02	0.01	0.12	2.35	0.019*
Substance use severity	−0.04	0.02	−0.11	−2.20	0.028*
Age at MRI scan	−0.05	0.04	−0.06	−1.20	0.230
Brain volume	0.00	0.00	0.01	0.29	0.769

Regression #1 with the dependent measure being loading coefficients obtained from SBM Component 6: R^2^ = 0.023, *R* = 0.153, *F*(412) = 2.44, *p* = 0.046; Regression #2 with the dependent measure being loading coefficients obtained from SBM Component 12: R^2^ = 0.052, *R* = 0.227, *F*(412) = 5.54, *p* < 0.001; Regression #3 with the dependent measure being loading coefficients obtained from SBM Component 28: R^2^ = 0.053, *R* = 0.231, *F*(412) = 5.76, *p* < 0.001; Regression #4 with the dependent measure being loading coefficients obtained from SBM Component 29: R^2^ = 0.032, *R* = 0.180, *F*(412) = 3.43, *p* = 0.009.

*Indicates results *p* < 0.05. **Indicates results which survived family-wise Bonferroni multiple comparison correction (i.e., 0.05/4, or *p* < 0.0125).

## Discussion

The current study investigated the association between youth psychopathic traits, assessed via the PCL:YV, and gray matter metrics (i.e., loading coefficients) obtained from multivariate SBM analysis of sMRI data. We observed that PCL:YV total scores were associated with loading coefficients obtained from four SBM components, each comprised of hypothesized limbic and paralimbic regions. Additionally, each of these four SBM components associated with PCL:YV total scores included additional regions extending beyond limbic and paralimbic regions, including frontal, parietal, and temporal/occipital brain regions. Our results extend upon previously published studies, identifying deficits within large-scale structural networks using multivariate SBM analyses among youth with elevated psychopathic traits.

Compared to youth scoring low on the PCL:YV, we observed that youth scoring high on the PCL:YV contributed less (i.e., exhibited reduced GMV) to large-scale structural networks containing limbic and paralimbic regions outlined in prior neurobiological theories (Blair, 2003, 2007, 2013; Kiehl, 2006), including the OFC, amygdala, insula, temporal pole, parahippocampal gyrus, and PCC. Additionally, each of these large-scale structural networks contained additional regions extending beyond limbic and paralimbic regions, including the SFG, MFG, IFG, IPL, supramarginal gyrus, precentral gyrus, fusiform gyrus, and cerebellum. These results suggest that rather than being circumscribed to deficits within limbic and paralimbic regions, youth with elevated psychopathic traits may instead be characterized by widespread structural deficits, spanning from limbic and paralimbic regions, to frontal, parietal, temporo-occipital, and cerebellar regions.

Prior studies incorporating univariate statistical analyses most commonly report structural abnormalities within limbic and paralimbic regions among youth with elevated psychopathic traits (Aghajani et al., 2017; Bashford-Largo et al., 2025; Bolhuis et al., 2019; Caldwell et al., 2019; Cohn et al., 2016; Cope et al., 2014; De Brito et al., 2009; Ermer et al., 2013; Fairchild et al., 2013; Gao et al., 2024; Sebastian et al., 2016; Steele et al., 2017; Waller et al., 2020; Walters and Kiehl, 2015). However, youth with elevated psychopathic traits have also been infrequently associated with structural abnormalities in regions extending beyond limbic and paralimbic regions including the SFG (Bolhuis et al., 2019; Waller et al., 2020), MFG (Gao et al., 2024; Jiang et al., 2023; Sebastian et al., 2016), IPL (Ermer et al., 2013; Gao et al., 2024; Yang et al., 2015), precuneus (Caldwell et al., 2019; Gao et al., 2024), and fusiform gyrus (Bolhuis et al., 2019) when incorporating univariate statistical analyses. While similar structural abnormalities have been observed with prior univariate analyses and our current multivariate analyses, the current study is the first to identify deficits within large-scale structural networks among youth with elevated psychopathic traits. Compared to univariate approaches, multivariate sMRI analyses allow for structural connectivity to be measured between regions implicated among youth with elevated psychopathic traits (Gupta et al., 2019). These interconnections between brain regions contained within large-scale structural networks would otherwise be lost when solely incorporating traditional univariate analyses. Furthermore, multivariate SBM analyses allow for the identification of patterns that are not specifically hypothesized (Gupta et al., 2019). Consistent with this notion, we observed that PCL:YV total scores were associated with deficits within large-scale structural networks containing regions not commonly implicated among youth with elevated psychopathic traits, including the supramarginal gyrus, precentral gyrus, and specific subregions of the cerebellum, including the uvula and vermis. The use of multivariate SBM analyses therefore allows for the identification of large-scale structural networks containing both regions frequently (e.g., limbic and paralimbic regions) and infrequently (e.g., regions extending beyond limbic and paralimbic regions) related to youth with elevated psychopathic traits.

The results obtained in the current study have important implications for identifying youth at risk for life-course persistent offending. Compared to youth with low levels of psychopathic traits, youth with elevated psychopathic traits are believed to represent life-course persistent antisocial offenders (Lussier et al., 2022; Piquero et al., 2012). Youth with elevated psychopathic traits not only begin engaging in criminal activity at an earlier age compared to youth with low levels of psychopathic traits (Farrington and Bergstrøm, 2022; Pechorro et al., 2014), but continue to engage in antisocial behavior throughout their lifespan (i.e., into adulthood; Allen et al., 2024a,b; Farrington and Bergstrøm, 2022). Incorporating SBM, Kiehl et al. (2018) identified that loading coefficients obtained from several SBM components containing regions outlined in the current study, including the temporal pole, amygdala, and OFC, were predictive of future antisocial outcomes (i.e., recidivism) among incarcerated adult men. The results of Kiehl et al. (2018) suggest that individuals with reduced structural coherence within SBM components containing the temporal pole, amygdala, and insula were at higher risk of recidivism compared to individuals with higher structural coherence between these same brain regions. When applied to the current study, this suggests that youth exhibiting reduced structural coherence within similar SBM components might also be at an increased risk for life-long, persistent antisocial offending throughout the lifespan.

Identifying specific large-scale structural networks implicated among youth with elevated psychopathic traits may additionally lead to the development of specialized treatment modalities designed to improve outcomes for high-risk youth. For example, following focused treatment intervention approaches (e.g., cognitive behavioral therapy and mindfulness), increased structural connectivity has been observed between regions identified in the current study, including the amygdala and OFC (Steiger et al., 2016), insula and temporal pole (Fahmy et al., 2018), and between different frontal regions (e.g., the SFG, MFG, and IFG; Fahmy et al., 2018). Though speculative, youth elevated psychopathic traits might similarly benefit from such focused treatment intervention approaches, resulting in improved structural connectivity within large-scale structural networks (e.g., Fahmy et al., 2018), contributing to reduced rates of future antisocial behavior.

### Limitations

Several limitations associated with our study should be noted. First, participants included in the current study were all incarcerated boys. While prior research suggests that incarcerated boys and girls scoring high on the PCL:YV are characterized by similar structural abnormalities using univariate statistical analyses (Cope et al., 2014; Ermer et al., 2013), whether boys and girls with elevated psychopathic traits exhibit similar deficits within large-scale structural networks is unknown. Second, it is unknown whether our results extend to alternative samples beyond incarcerated youth, including youth with elevated psychopathic traits recruited from the general community. Results presented in our Supplementary material suggest that core personality traits measuring interpersonal and affective dysfunction (i.e., PCL:YV Facet 1 and 2 scores) were associated with loading coefficients obtained from SBM Components 6, 12, 28, and 29, rather than PCL:YV Facet 4 scores measuring antisocial behavior more generally. These results therefore suggest that similar abnormalities within large-scale structural networks would be observed, regardless of whether youth with elevated psychopathic traits were recruited from correctional facilities or the general community. Future studies should be conducted to explore whether youth with elevated psychopathic traits recruited from multiple samples are associated with similar structural abnormalities within large-scale structural networks. Additionally, we performed SBM analyses using 30 total components, to be consistent with prior research studies (Anderson et al., 2019; Kiehl et al., 2018; Maurer et al., 2025; Turner et al., 2012). However, whether our current results extend to SBM analyses conducted with an alternative number of total components chosen (i.e., either a higher or lower total number) remains an open question. Additionally, participants in our sample ranged from 13.82 to 20.76 years of age (*M* = 17.14 years, SD = 1.19). Given that a significant portion of our sample was over 18 in the “adult” age range, we decided to use the TPM.nii (adult) template for tissue segmentation. The choice of template (and algorithm) for tissue segmentation or spatial normalization will yield different results, sometimes drastically different results (see Calhoun et al., 2017; Wilke et al., 2002; Yoon et al., 2009). However, among adolescents and young adults, similar results have been observed when utilizing both age-specific and adult template TPMs (see McKay et al., 2026). Prior SBM studies in children and adolescents have used the default adult TPM (Liu et al., 2024; Shen et al., 2022; Wang et al., 2025). To be consistent with prior research, we therefore similarly incorporated SPM12’s default TPM for tissue segmentation. Nevertheless, there is clearly a need to conduct a study examining how different templates may alter results obtained in SBM studies. Finally, prior research suggests that large-scale structural networks obtained via SBM correspond closely to functional networks obtained via resting-state fMRI (Segall et al., 2012). Despite these similarities, we cannot speak as to whether the large-scale structural networks obtained in the current study contribute to specific cognitive processes or specific domains implicated among youth with elevated psychopathic traits. More research is desperately needed to identify the association between large-scale SBM structural networks and functional networks obtained during different experimental paradigms, including tasks measuring cognitive control, empathy, and affective processing.

## Conclusion

Using multivariate SBM analyses of sMRI data, we observed that youth with elevated psychopathic traits exhibited deficits within large-scale structural networks, containing both limbic and paralimbic regions (e.g., the OFC, amygdala, temporal pole, insula, ACC, PCC, parahippocampal gyrus) and additional regions extending beyond limbic and paralimbic regions (e.g., the SFG, MFG, IFG, precuneus, IPL, fusiform gyrus, supramarginal gyrus, and precentral gyrus). This is the first study to our knowledge to identify deficits within large-scale structural networks among youth with elevated psychopathic traits, further improving our understanding of widespread structural abnormalities among youth at risk for life-long, persistent antisocial behavior.

## Data Availability

Data in this manuscript are not readily available due to the potential for re-identification of participants in this sensitive population (i.e., incarcerated juvenile offenders). However, data may be shared under a data use agreement by contacting KK, kkiehl@mrn.org.

## Footnotes

1
https://www.fil.ion.ucl.ac.uk/spm/software/spm12/

2
https://github.com/lrq3000/auto_acpc_reorient/

3
https://trendscenter.org/software/gift/

4 When implementing a family-wise Bonferroni correction (i.e., 0.05/4, or *p* < 0.0125), three of the correlation analyses survived multiple comparison correction (i.e., correlations between PCL:YV total scores and loading coefficients obtained from SBM Components 6, 12, and 28). However, the correlation between PCL:YV total scores and loading coefficients from SBM Component 29 did not survive this multiple comparison correction (*p* = 0.022).

5 When implementing a modified Bonferroni correction (i.e., 0.05/4, or *p* < 0.0125), PCL:YV scores remained associated with loading coefficients from SBM Components 6, 12, and 28 in multiple regression ^snm^analyses. However, PCL:YV scores were not significantly associated with loading coefficients from SBM Component 29 after multiple comparison correction (i.e., *p* = 0.019).
