# Supplementary material for: Source-based morphometry analyses yield associations between large-scale structural brain networks and psychopathic traits in incarcerated adolescents
Source: Front Psychol. 2026 Sep 29;17:1884761. doi: 10.3389/fpsyg.2026.1884761 (PMC13657932; doi:10.3389/fpsyg.2026.1884761)
Supplement: Supplementary file 1 [file Data_Sheet_1.pdf]

<u>Supplemental Material</u>

<u>Correlations Between Study Variables Included in Multiple Regression Analyses</u>

Correlations are reported in Supplemental Table 1 (below) between adolescent psychopathy scores (i.e., total, factor, and facet scores from the PCL:YV), substance use severity scores (operationally defined using a modified version of the ASI), participant's age at MRI data collection, and total brain volume (i.e., a sum of each participant's total gray and white matter volume).

Supplemental Table 1

Variable	PCL:YV Total	PCL:YV Factor 1	PCL:YV Factor 2	PCL:YV Facet 1	PCL:YV Facet 2	PCL:YV Facet 3	PCL:YV Facet 4	Sub. Use.	Age at MRI Scan	Brain Volume
**PCL:YV Total**	____	.863**	.838**	.715**	.761**	.750**	.655**	.168**	-.193**	-.010
**PCL:YV Factor 1**		____	.488**	.871**	.835**	.467**	.346**	.013	-.184**	.022
**PCL:YV Factor 2**			____	.355**	.485**	.869**	.810**	.291**	-.172**	-.041
**PCL:YV Facet 1**				____	.456**	.391**	.191**	.009	-.125*	.064
**PCL:YV Facet 2**					____	.408**	.411**	.013	-.192**	-.032
**PCL:YV Facet 3**						____	.414**	.204**	-.182**	-.018
**PCL:YV Facet 4**							____	.295**	-.103*	-.055
**Sub. Use.**								____	.227**	.037
**Age at MRI Scan**									____	.010

**Brain Volume**	——

Note. **refers to significant correlations at $p < .001$ threshold; *refers to significant correlations at $p < .05$ threshold.

Correlations Between PCL:YV Factor and Facet Scores and Loading Coefficients Obtained From SBM Components 6, 12, 28, and 29

In our main manuscript, we conducted correlation analyses between PCL:YV total scores and loading coefficients obtained from SBM Components 6, 12, 28, and 29. Here, we perform similar correlation analyses, replacing PCL:YV total scores with PCL:YV factor or facet scores.

Correlations Between PCL:YV Factor and Facet Scores with SBM Component 6 Loading Coefficients

Loading coefficients obtained from SBM Component 6 were positively correlated with PCL:YV Factor 1 scores (r = .12, p = .019), PCL:YV Factor 2 scores (r = .13, p = .009), PCL:YV Facet 1 scores (r = .10, p = .048), PCL:YV Facet 2 scores (r = .10, p = .044), PCL:YV Facet 3 scores (r = .10, p = .037), and PCL:YV Facet 4 scores (r = .11, p = .021).

Correlations Between PCL:YV Factor and Facet Scores with SBM Component 12 Loading Coefficients

Loading coefficients obtained from SBM Component 12 were positively with PCL:YV Factor 1 scores (r = .13, p = .010), PCL:YV Facet 1 scores (r = .12, p = .019), PCL:YV Facet 2 scores (r = .10, p = .040), and PCL:YV Facet 3 scores (r = .10, p = .049). Loading coefficients obtained from SBM Component 12 were not significantly correlated with PCL:YV Factor 2 scores (r = .06, p = .236) or PCL:YV Facet 4 scores (r = -.01, p = .874).

Correlations Between PCL:YV Factor and Facet Scores with SBM Component 28 Loading Coefficients

Loading coefficients obtained from SBM Component 28 were positively correlated with PCL:YV Factor 1 scores (r = .12, p = .016), PCL:YV Factor 2 scores (r = .12, p = .017), PCL:YV Facet 2 scores (r = .13, p = .009), and PCL:YV Facet 3 scores (r = .12, p = .012). Loading coefficients obtained from SBM Component 28 were not significantly correlated with PCL:YV Facet 1 scores (r = .08, p = .125) or PCL:YV Facet 4 scores (r = .07, p = .156).

Correlations Between PCL:YV Factor and Facet Scores with SBM Component 29 Loading Coefficients

Loading coefficients obtained from SBM Component 29 were positively correlated with PCL:YV Factor 1 scores (r = .11, p = .021) and PCL:YV Facet 1 scores (r = .14, p = .004). Loading coefficients from SBM Component 29 were moderately correlated with PCL:YV Factor 2 scores (r = .09, p = .074) and PCL:YV Facet 3 scores (r = .08, p = .096). Loading coefficients obtained from SBM Component 29 were not significantly correlated with PCL:YV Facet 2 scores (r = .05, p = .353) or PCL:YV Facet 4 scores (r = .07, p = .187).

Multiple Regression Analyses with PCL:YV Factor and Facet Scores and Loading Coefficients Obtained From SBM Components 6, 12, 28, and 29

First, we investigated whether PCL:YV factor scores were significantly associated with loading coefficients obtained from SBM Components 6, 12, 28, and 29 with supplemental multiple regression analyses. Here, PCL:YV factor scores (i.e., PCL:YV Factor 1 and 2 scores) along with covariate measures (i.e., substance use severity scores, participant's age at MRI data collection, and total brain volume) were entered as simultaneous predictor variables. Loading coefficients from SBM Component 6 (Supplemental Regression #1), SBM Component 12 (Supplemental Regression #2), SBM Component 28 (Supplemental Regression #3), and SBM Component 29 (Supplemental Regression #4) were entered as dependent measures.

In Supplemental Regression #1, PCL:YV Factor 2 scores were moderately associated with increased loading coefficients obtained from SBM Component 6 ($\beta = .11$, $p = .079$). In Supplemental Regression #2, PCL:YV Factor 1 scores were moderately associated with increased loading coefficients obtained from SBM Component 12 ($\beta = .10$, $p = .073$). In Supplemental Regression #3, PCL:YV Factor 2 scores were moderately associated with increased loading coefficients obtained from SBM Component 28 ($\beta = .11$, $p = .062$). In Supplemental Regression #4, PCL:YV factor scores were not significantly associated with loading coefficients obtained from SBM Component 29. See full statistics below in Supplemental Table 2 and see Supplemental Tables 4 – 7 for a full list of brain regions included within SBM Components 6, 12, 28, and 29.

Supplemental Table 2

Multiple Regression Analyses

Variable	B	SE(B)	β	*t*	*p*-value
Supplemental Regression #1: SBM Component 6					
PCL:YV Factor 1	0.02	0.02	.055	0.97	.333
PCL:YV Factor 2	0.04	0.02	.106	1.76	.079
Substance Use Severity	-0.01	0.02	-.032	-0.59	.557
Age at MRI Scan	-0.03	0.04	-.038	-0.72	.469
Brain Volume	0.00	0.00	.053	1.08	.280
Supplemental Regression #2: SBM Component 12					
PCL:YV Factor 1	0.03	0.02	.101	1.80	.073
PCL:YV Factor 2	0.01	0.02	.017	0.28	.782
Substance Use Severity	-0.02	0.02	-.055	-1.03	.306
Age at MRI Scan	-0.07	0.04	-.084	-1.62	.105
Brain Volume	0.00	0.00	.147	3.03	.003
Supplemental Regression #3: SBM Component 28					
PCL:YV Factor 1	0.02	0.02	.065	1.15	.251
PCL:YV Factor 2	0.04	0.02	.112	1.87	.062
Substance Use Severity	-0.04	0.02	-.118	-2.22	.027
Age at MRI Scan	-0.02	0.04	-.019	-0.36	.719
Brain Volume	-0.00	0.00	-.133	-2.74	.006
Supplemental Regression #4: SBM Component 29					
PCL:YV Factor 1	0.02	0.02	.064	1.13	.261
PCL:YV Factor 2	0.03	0.02	.082	1.36	.176
Substance Use Severity	-0.04	0.02	-.118	-2.20	.028
Age at MRI Scan	-0.05	0.04	-.058	-1.12	.264
Brain Volume	0.00	0.00	.015	0.31	.756

Note. Supplemental Regression #1 with the dependent measure being loading coefficients obtained from SBM Component 6: R^2 = .026, R = .160, $F(412)$ = 2.14, p = .060; Supplemental Regression #2 with the dependent measure being loading coefficients obtained from SBM Component 12: R^2 = .048, R = .220, $F(412)$ = 4.14, p = .001; Supplemental Regression #3 with the dependent measure being loading coefficients obtained from SBM Component 28: R^2 = .052, R = .228, $F(412)$ = 4.46, p = .001;

Supplemental Regression #4 with the dependent measure being loading coefficients obtained from SBM Component 29: R^2 = .034, R = .183, $F(412)$ = 2.83, p = .016.

Next, we investigated whether PCL:YV facet scores (i.e., PCL:YV Facet 1, 2, 3, and 4 scores) were significantly associated with loading coefficients obtained from SBM Components 6, 12, 28, and 29 in supplemental regression analyses. Simultaneous predictor variables included PCL:YV Facet 1, 2, 3, and 4 scores, along with substance use severity scores, participant's age at MRI data collection, and total brain volume. Dependent measures included loading coefficients from SBM Component 6 (Supplemental Regression #5), SBM Component 12 (Supplemental Regression #6), SBM Component 28 (Supplemental Regression #7), and SBM Component 29 (Supplemental Regression #8).

We observed that PCL:YV facet scores were not significantly associated with loading coefficients obtained from SBM Component 6 and SBM Component 12. Additionally, we observed that PCL:YV Facet 3 scores were moderately associated with increased loading coefficients obtained from SBM Component 28 ($\beta = .10$, $p = .089$. Finally, we observed that PCL:YV Facet 1 scores were significantly associated with increased loading coefficients obtained from SBM Component 29 ($\beta = .14$, $p = .014$). See full statistics below in Supplemental Table 3 and see Supplemental Tables 4 – 7 for a full list of brain regions included within SBM Components 6, 12, 28, and 29.

Supplemental Table 3

Multiple Regression Analyses

Variable	B	SE(B)	β	*t*	*p*-value
Supplemental Regression #5: SBM Component 6					
PCL:YV Facet 1	0.02	0.03	.048	0.83	.405
PCL:YV Facet 2	0.01	0.03	.019	0.31	.758
PCL:YV Facet 3	0.02	0.03	.040	0.68	.500
PCL:YV Facet 4	0.06	0.04	.089	1.52	.130
Substance Use Severity	-0.01	0.02	-.035	-0.64	.521
Age at MRI Scan	-0.03	0.04	-.039	-0.75	.452
Brain Volume	0.00	0.00	.053	1.08	.281
Supplemental Regression #6: SBM Component 12					
PCL:YV Facet 1	0.02	0.03	.050	0.87	.376
PCL:YV Facet 2	0.03	0.03	.062	1.04	.301
PCL:YV Facet 3	0.04	0.03	.075	1.27	.204
PCL:YV Facet 4	-0.04	0.04	-.060	-1.04	.301
Substance Use Severity	-0.02	0.02	-.048	-0.89	.373
Age at MRI Scan	-0.07	0.04	-.081	-1.57	.117
Brain Volume	0.00	0.00	.145	2.98	.003
Supplemental Regression #7: SBM Component 28					
PCL:YV Facet 1	0.00	0.03	.009	0.15	.880
PCL:YV Facet 2	0.04	0.03	.068	1.13	.260
PCL:YV Facet 3	0.05	0.03	.100	1.71	.089
PCL:YV Facet 4	0.02	0.04	.024	0.41	.684
Substance Use Severity	-0.04	0.02	-.114	-2.12	.034
Age at MRI	-0.01	0.04	-.016	-0.31	.758
Brain Volume	-0.00	0.00	-.132	-2.70	.007
Supplemental Regression #8: SBM Component 29					
PCL:YV Facet 1	0.07	0.03	.140	2.46	.014
PCL:YV Facet 2	-0.04	0.03	-.079	-1.31	.191
PCL:YV Facet 3	0.02	0.03	.038	0.65	.519
PCL:YV Facet 4	0.05	0.04	.086	1.47	.142
Substance Use Severity	-0.04	0.02	-.125	-2.33	.020
Age at MRI Scan	-0.05	0.04	-.064	-1.24	.217

Brain Volume	0.00	0.00	.007	0.15	.878

Note. Supplemental Regression #5 with the dependent measure being loading coefficients from SBM Component 6: R^2 = .027, R = .163, *F*(412) = 1.58, *p* = .141; Supplemental Regression #6 with the dependent measure being loading coefficients from SBM Component 12: R^2 = .053, R = .231, *F*(412) = 3.36, *p* = .002; Supplemental Regression #7 with the dependent measure being loading coefficients from SBM Component 28: R^2 = .054, R = .232, *F*(412) = 3.28, *p* = .002; Supplemental Regression #8 with the dependent measure being loading coefficients from SBM Component 29: R^2 = .045, R = .213, *F*(412) = 276, *p* = .008.

Supplemental Table 4

List of Brain Regions Included Within SBM Component 6

Area	Brodmann Area	Left (Max Z)	MNI Left (x, y, z)	Right (Max Z)	MNI Right (x, y, z)
**Positive (Regions in Red)**					
Anterior Cingulate	10, 24, 25, 32, 33	7.9	(-3, 32, -2)	8.1	(6, 33, 0)
Caudate	*			1.1	(6, 9, -2)
Cerebellar Tonsil	*	1.2	(-33, -53, -34)	1.2	(34, -51, -33)
Cingulate Gyrus	23, 24, 31, 32	6.1	(-9, 15, 28)	6.0	(10, 22, 27)
Cuneus	18	1.5	(-22, -82, 18)	1.1	(13, -86, 24)
Declive	*	1.2	(-37, -81, -17)		
Fusiform Gyrus	18, 20	1.9	(-42, -28, -15)	1.8	(42, -27, -15)
Inferior Frontal Gyrus	47	2.3	(-36, 6, 30)	2.1	(37, 29, 0)
Inferior Parietal Lobule	40	2.2	(-37, -29, 39)	2.8	(37, -31, 42)
Inferior Semi-Lunar Lobule	*	1.4	(-3, -60, -42)	1.4	(4, -65, -42)
Inferior Temporal Gyrus	20			1.0	(42, -12, -30)
Insula	13	1.1	(-31, 25, 2)	1.2	(45, -38, 19)
Lingual Gyrus	18, 19	1.6	(-21, -68, 0)	1.3	(19, -68, 1)
Medial Frontal Gyrus	6, 8, 9, 10, 11, 25, 32	5.7	(-9, 35, -7)	4.3	(13, 40, 13)
Middle Frontal Gyrus	6, 9, 10, 11	1.8	(-36, 9, 33)	1.5	(34, 33, -4)
Middle Occipital Gyrus	19	1.6	(-24, -85, 15)	1.2	(30, -81, 19)
Middle Temporal Gyrus	19, 39	1.4	(-48, -28, -4)	2.7	(39, -60, 15)
Paracentral Lobule	5	1.2	(-9, -11, 43)	1.6	(16, -38, 48)
Parahippocampal Gyru	30			1.4	(24, -46, 5)
Postcentral Gyrus	2	1.3	(-37, -28, 44)	3.1	(37, -29, 39)
Posterior Cingulate	30, 31	2.1	(-19, -55, 12)	1.8	(21, -55, 11)
Precentral Gyrus	6	1.5	(-37, 1, 29)	1.6	(30, -10, 57)
Precuneus	31	2.9	(-25, -61, 36)	1.4	(13, -39, 45)
Pyramis	*	1.1	(-27, -67, -31)		
Rectal Gyrus	*	1.1	(-1, 27, -19)		
Subcallosal Gyrus	25	3.5	(-3, 23, -11)	3.2	(3, 23, -11)
Superior Frontal Gyrus	6, 9	1.4	(-12, 19, 61)	1.5	(7, 15, 48)
Superior Parietal Lobule	7	1.7	(-22, -60, 44)		
Superior Temporal Gyrus	22, 41	1.6	(-52, 10, -2)	2.0	(46, -37, 12)

Tuber	*	1.1	(-42, -78, -23)		
**Negative (Regions in Blue)**					
Angular Gyrus	39	2.1	(-39, -57, 32)	1.9	(40, -54, 32)
Caudate	*	1.1	(-15, 16, 5)	1.1	(16, 15, 2)
Cerebellar Tonsil	*	1.8	(-46, -45, -34)	1.7	(43, -44, -39)
Cingulate Gyrus	31	2.3	(-13, -54, 28)		
Culmen	*	1.7	(-39, -36, -25)	1.9	(40, -36, -25)
Cuneus	7, 17, 23, 30	1.2	(-9, -83, 10)	1.6	(15, -90, 9)
Declive	*			1.3	(9, -81, -20)
Fusiform Gyrus	19, 20, 36, 37	1.4	(-50, -39, -22)	1.5	(48, -39, -22)
Inferior Frontal Gyrus	9, 10, 11, 13, 44, 45, 46, 47	3.0	(-37, 34, 11)	3.2	(36, 35, 11)
Inferior Occipital Gyrus	*	1.1	(-33, -73, -6)	1.2	(33, -76, -4)
Inferior Parietal Lobule	2, 40	2.0	(-40, -48, 38)	2.0	(48, -39, 41)
Inferior Semi-Lunar Lobule	*	1.2	(-48, -64, -36)	2.2	(7, -75, -35)
Inferior Temporal Gyrus	20	2.0	(-53, -31, -15)	1.4	(55, -23, -17)
Insula	13	2.7	(-40, 14, 12)	1.8	(42, 14, 10)
Lentiform Nucleus	*	1.5	(-18, 17, -2)	1.2	(22, 16, -2)
Lingual Gyrus	*	1.6	(-27, -79, -5)	1.5	(33, -72, -4)
Medial Frontal Gyrus	6	1.8	(-19, 5, 48)	1.5	(22, 11, 45)
Middle Frontal Gyrus	6, 8, 9, 10, 11, 46	2.4	(-27, 4, 43)	3.0	(39, 38, 11)
Middle Occipital Gyrus	18, 37	1.2	(-30, -79, -7)	1.4	(37, -75, 16)
Middle Temporal Gyrus	21, 38, 39	2.1	(-53, -34, -12)	2.3	(58, -32, -5)
Paracentral Lobule	5, 6, 31	1.5	(-1, -25, 45)	1.8	(9, -24, 48)
Parahippocampal Gyrus	19, 36	1.2	(-31, -42, -4)	1.1	(31, -11, -26)
Postcentral Gyrus	1, 2, 3, 5, 40, 43	1.9	(-42, -34, 57)	2.0	(39, -36, 60)
Posterior Cingulate	31	1.6	(-9, -54, 25)		
Precentral Gyrus	6, 44	3.5	(-43, 15, 9)	1.9	(45, 13, 8)
Precuneus	7, 19, 31, 39	2.6	(-12, -57, 30)	2.4	(25, -47, 47)
Pyramis	*	2.1	(-4, -77, -30)	2.7	(4, -77, -30)
Pyramis of Vermis	*	2.7	(0, -76, -30)	2.3	(1, -71, -30)
Rectal Gyrus	*			1.3	(16, 11, -21)
Superior Frontal Gyrus	8, 9, 10	1.9	(-21, 50, 13)	2.4	(22, 45, 21)

Superior Occipital Gyrus	19			1.6	(36, -73, 26)
Superior Parietal Lobule	7	1.4	(-27, -50, 44)	1.5	(33, -48, 51)
Superior Temporal Gyrus	38, 39, 42	1.9	(-42, -57, 29)	1.8	(45, -55, 29)
Supramarginal Gyrus	40	3.3	(-40, -49, 30)	2.9	(43, -51, 30)
Tuber	*	1.2	(-50, -48, -24)		
Tuber of Vermis	*	2.3	(0, -77, -26)		
Uncus	*			1.3	(22, 8, -23)
Uvula	*	2.4	(-3, -74, -33)	2.3	(7, -77, -33)
Uvula of Vermis	*	2.8	(0, -72, -34)	2.2	(3, -69, -33)

Note. Regions listed above are those included within SBM Component 6. This list of brain regions was determined via the GIFT toolbox with the following parameters: (1) images were converted to Z-scores, (2) a Z-score threshold of > 1.0 was used, and (3) the distance between contiguous voxels in mm was set to 4.

Supplemental Table 5

List of Brain Regions Included Within SBM Component 12

Area	Brodmann Area	Left (Max Z)	MNI Left (x, y, z)	Right (Max Z)	MNI Right (x, y, z)
**Positive (Regions in Red)**					
Angular Gyrus	*	1.3	(-42, -66, 30)		
Anterior Cingulate	10, 25, 32	1.1	(0, 7, -9)	1.1	(10, 49, -1)
Cerebellar Tonsil	*	2.7	(-42, -43, -34)	2.4	(40, -43, -36)
Claustrum	*	1.1	(-37, -19, 1)	1.3	(34, -19, 6)
Culmen	*	2.4	(-43, -42, -26)	2.7	(46, -44, -27)
Cuneus	17, 18	2.1	(-12, -95, 0)	1.4	(6, -81, 14)
Declive	*	1.3	(-7, -83, -22)	1.0	(48, -52, -21)
Fusiform Gyrus	20, 36, 37	1.7	(-50, -40, -22)	1.9	(50, -40, -22)
Inferior Frontal Gyrus	9, 45, 46, 47	1.7	(-50, 4, 25)	1.6	(48, 3, 30)
Inferior Occipital Gyrus	17, 18, 19	1.6	(-18, -91, -8)	1.5	(34, -75, -5)
Inferior Parietal Lobule	7, 40	2.4	(-42, -32, 24)	1.6	(46, -31, 25)
Inferior Semi-Lunar Lobule	*	1.9	(-7, -75, -38)		
Inferior Temporal Gyrus	19, 20, 37	1.7	(-55, -46, -13)	2.0	(55, -26, -16)
Insula	13, 22, 40	3.0	(-40, -35, 21)	2.2	(39, -30, 21)
Lentiform Nucleus	*	1.7	(-25, -3, 6)	1.3	(27, -3, 6)
Lingual Gyrus	17, 18	2.2	(-12, -92, -3)	1.3	(34, -72, -8)
Medial Frontal Gyrus	10			1.2	(10, 49, -5)
Middle Frontal Gyrus	6, 8, 9, 10, 46	1.8	(-40, 23, 28)	2.9	(27, 2, 45)
Middle Occipital Gyrus	*	1.3	(-43, -69, -7)	1.5	(22, -90, 9)
Middle Temporal Gyrus	20, 21, 22, 37, 38, 39	2.1	(-42, -60, 24)	2.8	(49, -43, 8)
Paracentral Lobule	*	1.0	(-7, -20, 45)	1.1	(9, -30, 50)
Parahippocampal Gyrus	19, 36, 37	1.7	(-31, -37, -4)	1.5	(33, -38, -4)
Postcentral Gyrus	2, 3, 4, 40, 43	1.4	(-24, -31, 58)	1.6	(25, -29, 59)
Precentral Gyrus	4, 6, 9, 13, 44	1.9	(-48, 3, 27)	2.1	(28, -25, 59)
Precuneus	7, 31	2.0	(-24, -58, 39)	1.4	(13, -58, 35)
Pyramis	*	1.8	(-3, -79, -26)	1.4	(4, -77, -30)
Pyramis of Vermis	*	1.1	(-1, -74, -2)	1.6	(1, -77, -28)
Subcallosal Gyrus	25	1.0	(-3, 4, -14)		
Superior Frontal Gyrus	8, 9, 10	1.7	(-22, 45, 24)	1.5	(28, 13, 49)

Superior Parietal Lobule	7	1.6	(-24, -59, 46)	1.0	(28, -55, 44)
Superior Temporal Gyrus	13, 22, 39, 41, 42	2.5	(-39, -36, 17)	2.6	(46, -45, 11)
Thalamus	*	1.2	(-3, -20, 7)	1.2	(3, -17, 4)
Transverse Temporal Gyrus	41, 42	1.1	(-37, -27, 11)	1.4	(62, -15, 13)
Tuber	*	1.2	(-52, -48, -23)	1.9	(48, -49, -24)
Tuber of Vermis	*	1.2	(0, -77, -24)		
Uncus	*	1.3	(-33, -1, -28)		
Uvula	*	2.0	(-6, -76, -34)		
**Negative (Regions in Blue)**					
Cerebellar Tonsil	*	1.6	(-46, -63, -41)	1.2	(31, -48, -32)
Cingulate Gyrus	23	1.4	(-10, -20, 34)	1.3	(6, -22, 27)
Culmen	*			1.4	(27, -48, -25)
Cuneus	7, 17, 18, 19	1.1	(-12, -81, 8)	1.6	(15, -86, 21)
Declive	*	2.2	(-33, -86, -22)		
Fusiform Gyrus	20, 37	3.8	(-42, -25, -25)	4.0	(43, -27, -16)
Inferior Frontal Gyrus	9, 11, 13, 44, 47	2.4	(-34, 9, 29)	1.9	(40, 17, -18)
Inferior Parietal Lobule	40	1.3	(-46, -35, 36)	1.5	(48, -39, 38)
Inferior Semi-Lunar Lobule	*	2.4	(-36, -78, -35)	2.3	(25, -82, -35)
Inferior Temporal Gyrus	20, 21	7.0	(-30, -2, -40)	6.7	(31, -2, -42)
Insula	13	1.0	(-37, 21, 10)	1.5	(52, -38, 19)
Medial Frontal Gyrus	11	1.4	(-12, 62, -16)	1.2	(6, 62, -17)
Middle Frontal Gyrus	6, 8, 9, 10, 11, 47	2.7	(-33, 18, 38)	2.4	(33, 11, 38)
Middle Temporal Gyrus	20, 21, 38, 39	8.1	(-31, 2, -40)	7.3	(37, 10, -38)
Orbital Gyrus	11, 47	1.5	(-9, 38, -27)	1.9	(7, 41, -27)
Parahippocampal Gyrus	28, 34, 35, 36	2.3	(-19, -7, -25)	3.3	(40, -27, -19)
Postcentral Gyrus	*	1.4	(-55, -15, 23)		
Posterior Cingulate	*	1.8	(-13, -51, 19)		
Precentral Gyrus	6, 9	2.6	(-33, 15, 35)	1.6	(34, 9, 34)
Precuneus	7, 31	1.4	(-13, -47, 55)	1.8	(21, -69, 26)
Pyramis	*	2.6	(-34, -80, -33)	2.2	(22, -83, -32)
Rectal Gyrus	11	1.5	(-7, 44, -26)	2.0	(7, 45, -26)
Subcallosal Gyrus	*	1.2	(-22, 11, -14)		

Superior Frontal Gyrus	6, 10, 11	2.0	(-19, 39, -16)	2.0	(10, 57, -22)
Superior Temporal Gyrus	22, 38, 39	8.9	(-28, 10, -38)	8.8	(30, 8, -40)
Supramarginal Gyrus	40	2.5	(-45, -45, 33)	2.0	(45, -39, 35)
Tuber	*	2.7	(-31, -83, -30)	1.6	(24, -84, -29)
Uncus	20, 28, 34, 36, 38	7.8	(-27, 1, -40)	7.6	(28, 1, -42)
Uvula	*	2.6	(-28, -86, -26)	1.2	(36, -80, -24)

Note. Regions listed above are those included within SBM Component 12. This list of brain regions was determined via the GIFT toolbox with the following parameters: (1) images were converted to Z-scores, (2) a Z-score threshold of > 1.0 was used, and (3) the distance between contiguous voxels in mm was set to 4.

Supplemental Table 6

List of Brain regions Included Within SBM Component 28

Area	Brodmann Area	Left (Max Z)	MNI Left (x, y, z)	Right (Max Z)	MNI Right (x, y, z)
**Positive (Regions in Red)**					
Angular Gyrus	*	1.4	(-42, -60, 31)	1.3	(46, -56, 36)
Anterior Cingulate	*	1.0	(-15, 46, 9)	1.0	(1, 5, -10)
Caudate	*	1.5	(-15, 11, 15)	1.4	(16, 14, 12)
Cerebellar Tonsil	*			1.3	(33, -60, -31)
Cingulate Gyrus	24, 31, 32	2.1	(-15, -36, 40)	2.1	(18, -53, 26)
Culmen	*	1.2	(-49, -46, -22)	1.8	(43, -36, -23)
Cuneus	17	2.0	(-25, -78, 19)	1.9	(24, -78, 20)
Declive	*	1.7	(-12, -87, -18)		
Declive of Vermis	*	1.0	(-1, -78, -19)	1.4	(1, -77, -21)
Fusiform Gyrus	19, 20, 36, 37	2.1	(-53, -49, -16)	2.2	(52, -38, -22)
Inferior Frontal Gyrus	9, 11, 44, 46, 47	2.4	(-45, 6, 23)	2.1	(37, 10, 26)
Inferior Occipital Gyrus	19	2.1	(-37, -72, -4)		
Inferior Parietal Lobule	40	1.7	(-43, -47, 24)	3.2	(49, -37, 38)
Inferior Temporal Gyrus	19, 20, 21, 37	2.3	(-56, -46, -15)	2.0	(59, -46, -17)
Insula	13, 22	2.3	(-42, -23, 0)	4.0	(48, -40, 20)
Lingual Gyrus	18	1.4	(-33, -72, -3)	1.8	(21, -82, 0)
Medial Frontal Gyrus	10, 25	1.9	(-21, 47, 9)	2.2	(24, 43, 17)
Middle Frontal Gyrus	6, 8, 10	2.1	(-24, -8, 49)	2.0	(34, 47, -2)
Middle Occipital Gyrus	18, 19, 37	2.4	(-27, -84, 4)	1.4	(27, -78, 18)
Middle Temporal Gyrus	19, 20, 21, 22, 37, 39	14.1	(-48, -35, 2)	7.2	(46, -25, -3)
Paracentral Lobule	5, 31	1.6	(0, -42, 59)	2.0	(3, -45, 58)
Parahippocampal Gyrus	28, 34, 36, 37	1.2	(-30, -40, -7)	1.2	(31, -35, -10)
Postcentral Gyrus	5			1.4	(6, -46, 64)
Posterior Cingulate	30	1.3	(-22, -64, 9)	1.1	(15, -50, 20)
Precentral Gyrus	4, 6	2.0	(-39, 3, 26)	1.9	(52, 7, 13)
Precuneus	7, 19, 31	2.4	(-30, -46, 48)	1.6	(15, -56, 26)
Pyramis	*	1.3	(-4, -80, -26)	1.4	(3, -77, -25)
Pyramis of Vermis	*	1.7	(0, -77, -28)	1.0	(3, -74, -28)
Rectal Gyrus	*	1.0	(-7, 11, -22)	1.4	(12, 13, -22)
Subcallosal Gyrus	25	1.2	(-9, 7, -14)	1.1	(4, 4, -13)

Superior Frontal Gyrus	6, 10	2.5	(-24, 47, 6)	2.5	(24, 40, 20)
Superior Parietal Lobule	7	1.4	(-27, -49, 43)	1.2	(34, -72, 45)
Superior Temporal Gyrus	13, 21, 22, 38, 39, 41	12.4	(-48, -26, -2)	9.2	(46, -38, 7)
Supramarginal Gyrus	40	3.0	(-45, -51, 23)	2.8	(49, -38, 34)
Thalamus	*	1.3	(-4, -15, 1)	1.4	(4, -15, 1)
Transverse Temporal Gyrus	*			2.2	(43, -32, 10)
Tuber	*			1.1	(30, -63, -30)
Tuber of Vermis	*	1.2	(0, -74, -25)		
Uncus	34			1.1	(15, -4, -20)
**Negative (Regions in Blue)**					
Angular Gyrus	39	3.6	(-31, -57, 35)	2.3	(34, -55, 37)
Anterior Cingulate	24	1.1	(-1, 35, 5)	1.1	(3, 35, 4)
Cerebellar Tonsil	*	1.1	(-46, -60, -39)		
Cingulate Gyrus	31			1.5	(10, -38, 32)
Claustrum	*	2.2	(-30, 8, -5)	3.1	(31, 10, -6)
Culmen	*	1.4	(-4, -57, -1)	1.1	(4, -60, -2)
Culmen of Vermis	*	1.3	(-3, -61, -2)	1.2	(4, -63, -4)
Cuneus	7, 18, 19	2.3	(-15, -71, 31)	1.7	(16, -94, 19)
Fusiform Gyrus	20, 37	2.9	(-39, -28, -15)	3.4	(40, -31, -15)
Inferior Frontal Gyrus	9, 10, 13, 44, 45, 46, 47	2.1	(-42, 28, 1)	2.5	(33, 28, 3)
Inferior Occipital Gyrus	17			1.3	(19, -92, -9)
Inferior Parietal Lobule	39, 40	3.0	(-33, -59, 38)	2.8	(34, -61, 38)
Inferior Temporal Gyrus	20	1.2	(-40, -16, -28)	1.2	(52, -54, -4)
Insula	13, 40, 41	4.4	(-40, -28, 14)	4.8	(39, -28, 14)
Lentiform Nucleus	*	1.5	(-24, 9, 5)	1.5	(25, 10, -6)
Lingual Gyrus	17, 19	1.3	(-21, -63, -2)	1.7	(19, -95, -4)
Medial Frontal Gyrus	6, 11	1.2	(-13, -6, 58)	1.4	(12, -6, 60)
Middle Frontal Gyrus	6, 9, 10, 11	2.0	(-19, -12, 59)	1.9	(36, 9, 40)
Middle Occipital Gyrus	18, 19	1.5	(-37, -75, 9)	2.3	(39, -77, 8)
Middle Temporal Gyrus	21, 39	2.8	(-48, -54, 4)	2.0	(49, -54, -1)
Paracentral Lobule	5	1.4	(-19, -44, 57)		
Parahippocampal Gyrus	30, 36	3.3	(-39, -20, -17)	3.1	(40, -23, -17)

Postcentral Gyrus	1, 2, 3, 5, 40, 43	2.7	(-53, -24, 14)	3.8	(39, -26, 39)
Posterior Cingulate	29	1.5	(-7, -46, 16)	1.1	(10, -43, 8)
Precentral Gyrus	3, 6, 9, 13, 43, 44	2.5	(-49, -15, 12)	2.8	(48, -16, 10)
Precuneus	7, 19, 31	3.9	(-30, -61, 35)	2.7	(36, -64, 34)
Rectal Gyrus	*			1.1	(7, 44, -26)
Superior Frontal Gyrus	6, 8, 10	1.2	(-19, -14, 63)	1.6	(16, 45, 28)
Superior Occipital Gyrus	19			1.1	(36, -74, 27)
Superior Parietal Lobule	5, 7	1.8	(-22, -44, 60)	2.0	(30, -56, 43)
Superior Temporal Gyrus	13, 21, 22, 38, 41, 42	5.2	(-39, -29, 8)	3.8	(48, -20, 9)
Supramarginal Gyrus	*	3.2	(-36, -51, 37)	1.2	(50, -51, 23)
Thalamus	*			1.0	(19, -30, 6)
Transverse Temporal Gyrus	41, 42	6.2	(-40, -31, 11)	4.5	(42, -26, 12)

Note. Regions listed above are those included within SBM Component 28. This list of brain regions was determined via the GIFT toolbox with the following parameters: (1) images were converted to Z-scores, (2) a Z-score threshold of > 1.0 was used, and (3) the distance between contiguous voxels in mm was set to 4.

Supplemental Table 7

List of Brain regions Included Within SBM Component 29

Area	Brodmann Area	Left (Max Z)	MNI Left (x, y, z)	Right (Max Z)	MNI Right (x, y, z)
**Positive (Regions in Red)**					
Angular Gyrus	39	2.9	(-42, -58, 31)	2.8	(43, -57, 30)
Anterior Cingulate	32	1.1	(-12, 41, -3)	1.6	(12, 36, 24)
Cerebellar Tonsil	*	1.5	(-9, -56, -33)		
Cingulate Gyrus	23, 24, 31	2.8	(-1, -29, 28)	2.8	(4, -26, 26)
Claustrum	*			1.1	(33, -13, 10)
Culmen	*	1.2	(-39, -40, -19)	1.0	(37, -42, -19)
Cuneus	7, 18, 19	4.1	(-21, -80, 30)	3.4	(27, -77, 33)
Fusiform Nucleus	19, 20, 36,37	4.5	(-40, -43, -12)	5.2	(43, -41, -12)
Inferior Frontal Gyrus	9, 13, 44, 45, 46, 47	3.2	(-34, 9, 29)	2.4	(42, 33, 11)
Inferior Occipital Gyrus	19	3.9	(-37, -69, -3)	1.9	(40, -70, -5)
Inferior Parietal Lobule	40	2.5	(-46, -46, 40)	2.0	(43, -45, 40)
Inferior Semi-Lunar Lobule	*	1.0	(-7, -75, -38)	1.3	(9, -71, -42)
Inferior Temporal Gyrus	19, 20, 37	6.5	(-42, -67, 2)	3.4	(45, -68, 2)
Insula	13	1.2	(-37, 22, 10)	1.3	(42, 18, 6)
Lentiform Nucleus	*	1.1	(-28, -10, 3)		
Lingual Gyrus	18, 19	2.8	(-28, -74, -4)	1.8	(22, -54, -2)
Medial Frontal Gyrus	10	1.4	(-13, 47, 3)	1.5	(21, 10, 46)
Middle Frontal Gyrus	6, 8, 9, 46	2.8	(-34, 12, 31)	3.6	(37, 10, 31)
Middle Occipital Gyrus	18, 19, 37	8.5	(-39, -64, 3)	8.6	(40, -64, 9)
Middle Temporal Gyrus	19, 21, 22, 37, 39	6.7	(-42, -67, 7)	7.0	(40, -62, 17)
Paracentral Lobule	*	1.0	(-10, -32, 51)		
Parahippocampal Gyrus	19, 34	1.3	(-39, -33, -12)	2.6	(40, -34, -13)
Postcentral Gyrus	2, 3, 5, 7, 40, 43	1.4	(-45, -18, 44)	1.5	(42, -20, 41)
Posterior Cingulate	23, 29, 30, 31	3.0	(-1, -32, 25)	2.9	(4, -32, 25)
Precentral Gyrus	6, 9, 44	2.2	(-34, 13, 35)	4.5	(34, 10, 34)
Precuneus	7, 19, 31, 39	4.2	(-12, -60, 32)	3.4	(25, -50, 47)
Superior Frontal Gyrus	8, 9, 10	1.5	(-19, 17, 43)	1.9	(27, 44, 16)
Superior Occipital Gyrus	19	1.1	(-39, -77, 27)	2.7	(30, -80, 28)
Superior Parietal Lobule	7	2.4	(-25, -50, 44)	2.6	(28, -50, 44)

Superior Temporal Gyrus	21, 22, 38, 39, 41, 42	3.2	(-39, -58, 28)	3.7	(40, -56, 17)
Supramarginal Gyrus	40	3.1	(-46, -46, 35)	3.1	(46, -42, 34)
Thalamus	*	2.3	(-9, -22, 16)	2.7	(9, -20, 16)
Transverse Temporal Gyrus	42	1.4	(-64, -16, 10)		
Uncus	28	1.4	(-13, -5, -20)		
Uvula	*	1.2	(-4, -77, -31)		
**Negative (Regions in Blue)**					
Angular Gyrus	39	2.9	(-34, -70, 31)	4.5	(34, -61, 36)
Anterior Cingulate	*	1.1	(0, 5, -9)		
Caudate	*			1.0	(36, -16, -8)
Cerebellar Lingual	*	2.5	(-3, -43, -7)	2.4	(1, -43, -7)
Cerebellar Tonsil	*	1.1	(-28, -40, -33)		
Cingulate Gyrus	24, 31, 32	2.4	(-15, -40, 42)	2.5	(16, -34, 42)
Culmen	*	2.6	(-1, -45, -3)	2.3	(3, -45, -3)
Culmen of Vermis	*	1.1	(-1, -65, -8)	1.2	(3, -65, -8)
Cuneus	7, 17, 18, 19, 23, 30	4.7	(-12, -73, 26)	4.8	(16, -70, 30)
Declive	*			1.7	(30, -83, -22)
Fusiform Gyrus	18, 19, 20, 37	1.8	(-56, -32, -20)	2.5	(56, -19, -26)
Inferior Frontal Gyrus	6, 9	2.2	(-45, 2, 32)	1.2	(43, 0, 32)
Inferior Occipital Gyrus	18			1.3	(33, -87, -13)
Inferior Parietal Lobule	39, 40	4.0	(-42, -33, 38)	3.4	(37, -33, 38)
Inferior Semi-Lunar Lobule	*	1.2	(-4, -63, -42)	2.1	(31, -79, -35)
Inferior Temporal Gyrus	20, 21	1.8	(-55, -16, -29)	2.2	(56, -15, -28)
Insula	13	1.2	(-46, -41, 19)	1.6	(45, -38, 20)
Lingual Gyrus	17, 18, 19	1.8	(-22, -55, 4)	2.4	(4, -88, -8)
Medial Frontal Gyrus	6, 8, 10, 25, 32	1.7	(-10, 15, 46)	1.4	(24, 35, 27)
Middle Frontal Gyrus	6, 8, 9, 11	4.2	(-28, 2, 43)	3.6	(30, 2, 44)
Middle Occipital Gyrus	18, 19	4.6	(-28, -75, 19)	1.9	(34, -72, 15)
Middle Temporal Gyrus	20, 21, 39	4.9	(-48, -49, 8)	4.6	(36, -67, 28)
Paracentral Lobule	5, 7	2.0	(-16, -40, 48)	2.3	(18, -40, 50)
Parahippocampal Gyrus	19, 30, 36, 37	2.2	(-33, -40, -6)	3.0	(22, -51, 5)
Postcentral Gyrus	2, 3, 5	2.3	(-48, -27, 37)	1.3	(43, -27, 37)
Posterior Cingulate	30, 31	3.0	(-18, -66, 17)	2.5	(22, -65, 9)
Precentral Gyrus	4, 6, 9	3.0	(-34, 2, 37)	2.7	(39, -1, 37)
Precuneus	7, 19, 23, 31, 39	5.4	(-28, -65, 35)	5.3	(34, -64, 34)

Pyramis	*			2.3	(30, -82, -32)
Rectal Gyrus	11	1.2	(-3, 28, -23)	1.3	(3, 27, -20)
Subcallosal Gyrus	25	1.0	(-3, 9, -14)	1.0	(3, 5, -12)
Superior Frontal Gyrus	6, 8	2.1	(-12, 16, 52)	2.0	(30, 17, 49)
Superior Occipital Gyrus	19			1.8	(36, -73, 29)
Superior Parietal Lobule	7	1.8	(-31, -66, 43)	2.1	(34, -60, 43)
Superior Temporal Gyrus	13, 22, 38, 39	7.1	(-43, -50, 14)	3.4	(45, -44, 16)
Supramarginal Gyrus	40	2.8	(-40, -38, 36)	1.3	(53, -48, 22)
Thalamus	*			1.4	(4, -16, 0)
Tuber	*			2.4	(33, -82, -30)
Uncus	20			1.3	(34, -16, -28)
Uvula	*			2.1	(33, -81, -25)

Note. Regions listed above are those included within SBM Component 29. This list of brain regions was determined via the GIFT toolbox with the following parameters: (1) images were converted to Z-scores, (2) a Z-score threshold of > 1.0 was used, and (3) the distance between contiguous voxels in mm was set to 4.

Investigating Brain Regions Included in SBM Components Using

Automated Anatomical Labeling 3 ROIs

In addition to identifying which brain regions were included within different SBM components through the Talairach table option available in the GIFT software package, we investigated the total number of SBM voxels contained within each of the 116 ROIs included within the Automated Anatomic Labelling Atlas 3 (Rolls et al., 2020), using different Z-score thresholds (i.e., Z > 1.0, 2.0, 3.0, and 4.0, respectively). Through this method, we present the AAL3 ROIs included within SBM Component 6 (Supplemental Figures 1 – 4), SBM Component 12 (Supplemental Figures 5 – 8), SBM Component 28 (Supplemental Figures 9 – 12), and SBM Component 29 (Supplemental Figures 13 – 16).

Supplemental Figure 1

AAL3 ROIs Included Within SBM Component 6

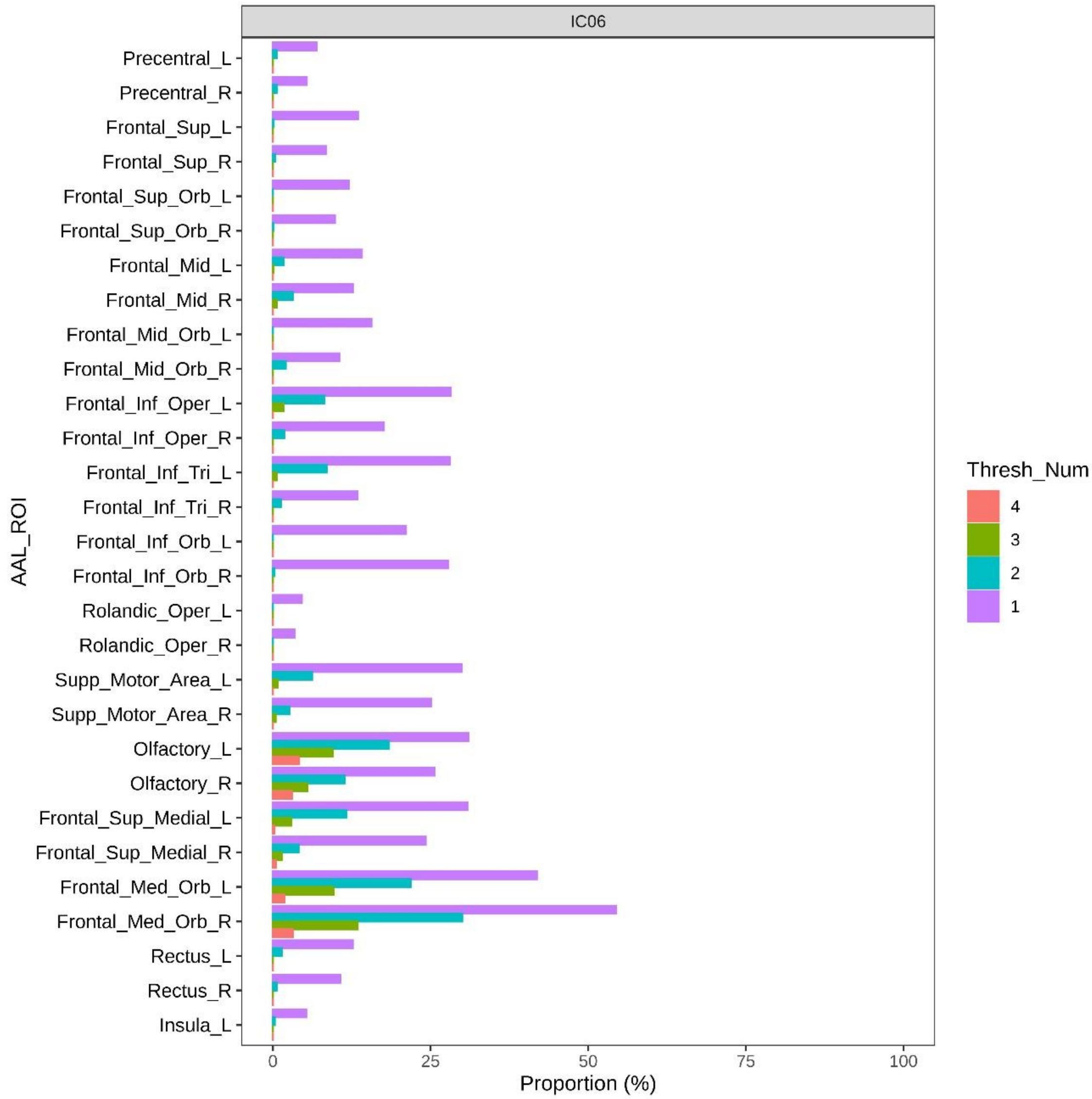

Note. Here, we list the first 29 ROIs included within the AAL3 atlas, determining whether or not these brain regions were included within SBM Component 6. The Y-axis lists the specific AAL3 ROIs and the X-axis displays the proportion of each AAL3 ROI included within SBM Component 6. Results displayed using a Z-threshold of 1.0 (purple), 2.0 (blue), 3.0 (green), and 4.0 (orange).

Supplemental Figure 2

AAL3 ROIs Included Within SBM Component 6

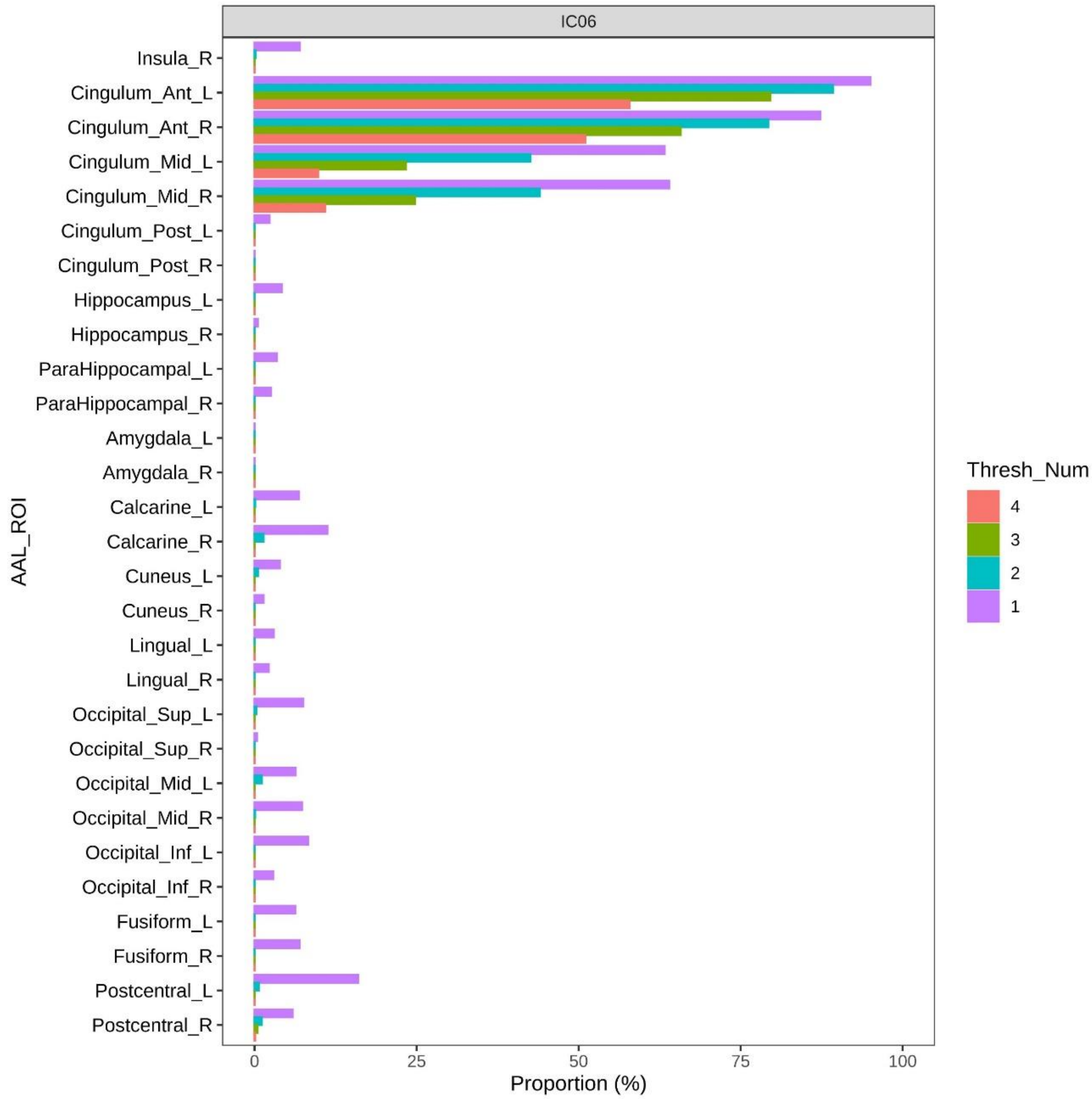

Note. Here, we list the next 29 ROIs included within the AAL3 atlas, determining whether or not these brain regions were included within SBM Component 6. The Y-axis lists the specific AAL3 ROIs and the X-axis displays the proportion of each AAL3 ROI included within SBM Component 6. Results displayed using a Z-threshold of 1.0 (purple), 2.0 (blue), 3.0 (green), and 4.0 (orange).

Supplemental Figure 3

AAL3 ROIs Included Within SBM Component 6

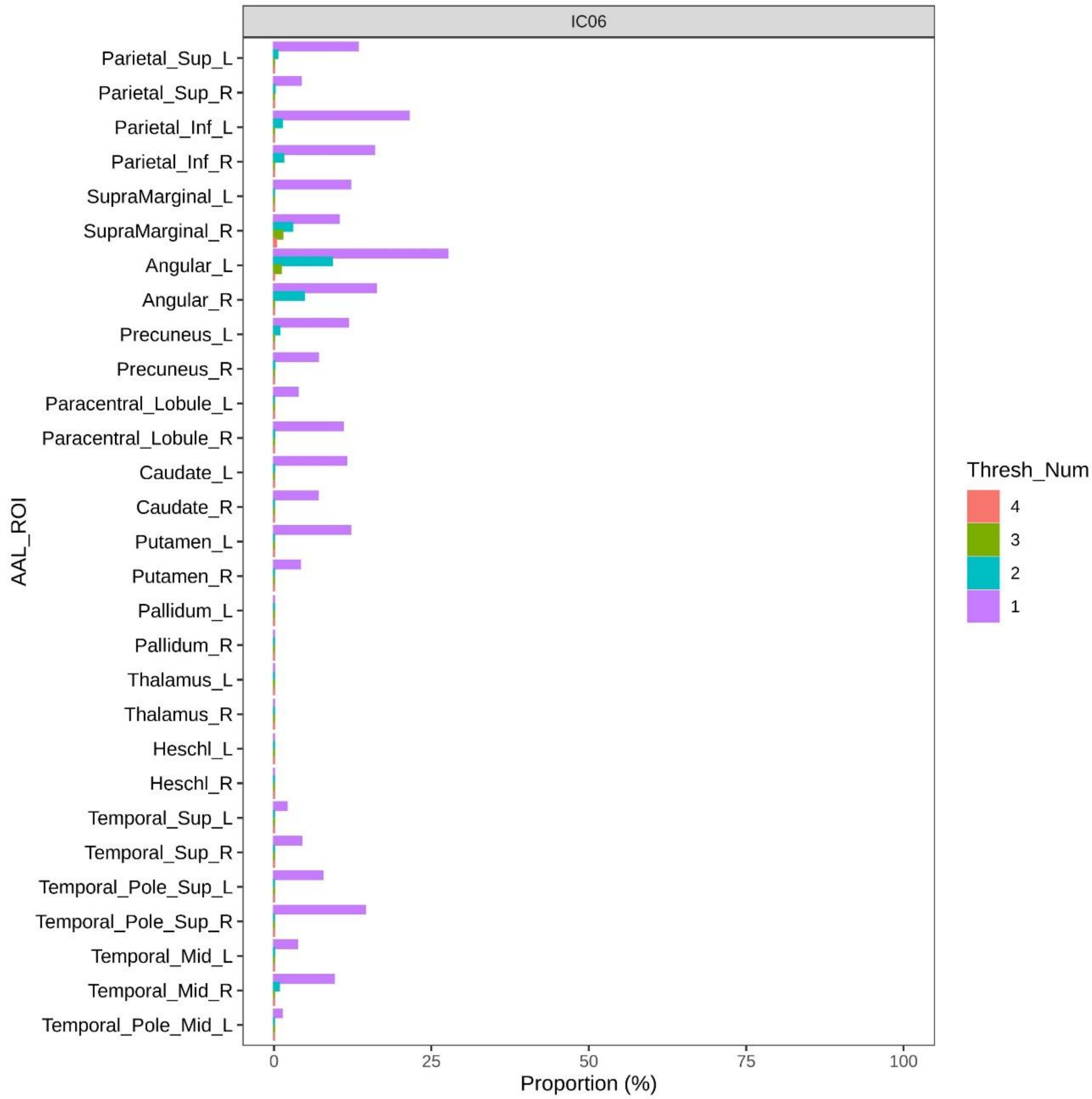

Note. Here, we list the next 29 ROIs included within the AAL3 atlas, determining whether or not these brain regions were included within SBM Component 6. The Y-axis lists the specific AAL3 ROIs and the X-axis displays the proportion of each AAL3 ROI included within SBM Component 6. Results displayed using a Z-threshold of 1.0 (purple), 2.0 (blue), 3.0 (green), and 4.0 (orange).

Supplemental Figure 4

AAL3 ROIs Included Within SBM Component 6

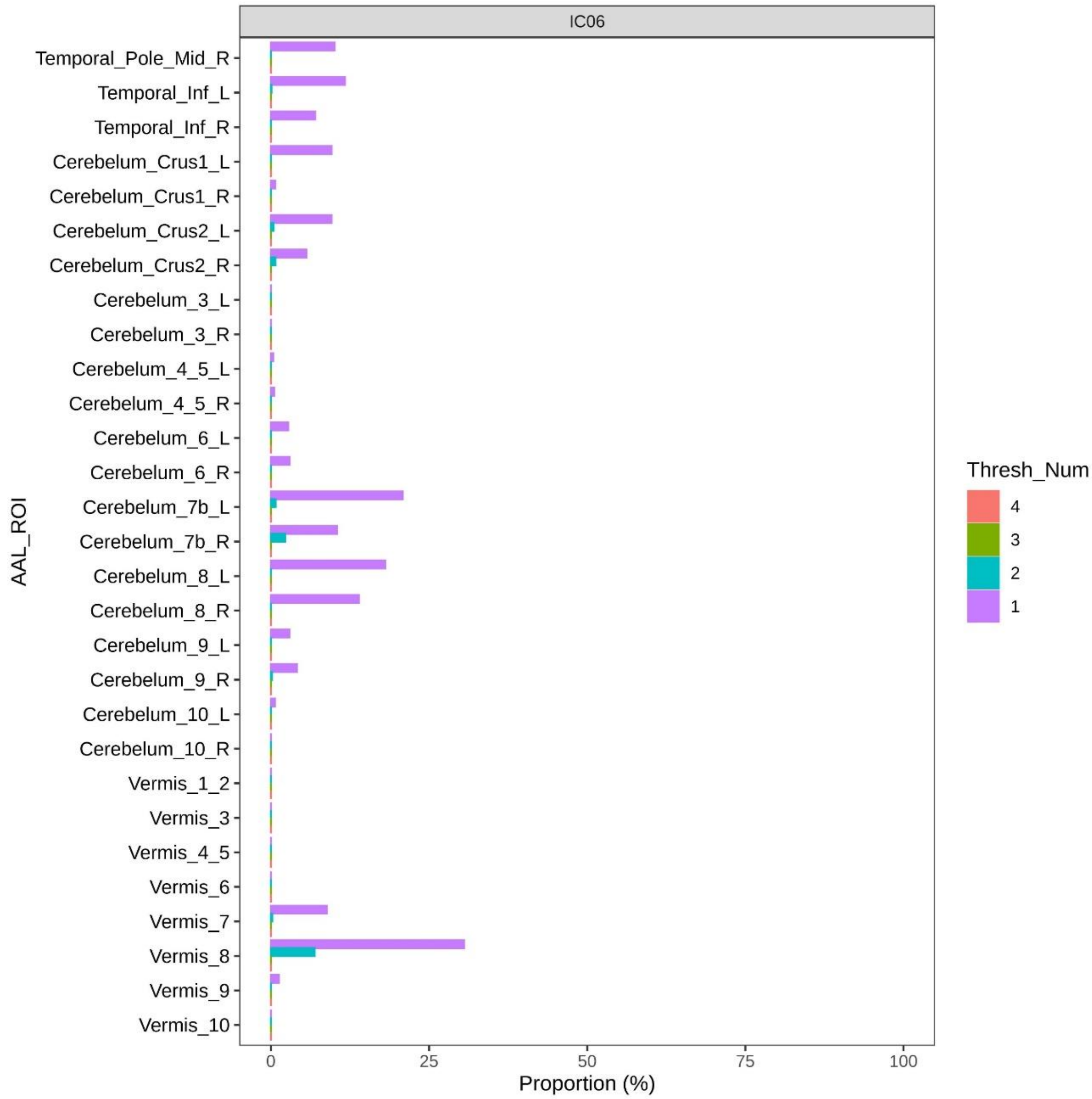

Note. Here, we list the final 29 ROIs included within the AAL3 atlas, determining whether or not these brain regions were included within SBM Component 6. The Y-axis lists the specific AAL3 ROIs and the X-axis displays the proportion of each AAL3 ROI included within SBM Component 6. Results displayed using a Z-threshold of 1.0 (purple), 2.0 (blue), 3.0 (green), and 4.0 (orange).

Supplemental Figure 5

AAL3 ROIs Included Within SBM Component 12

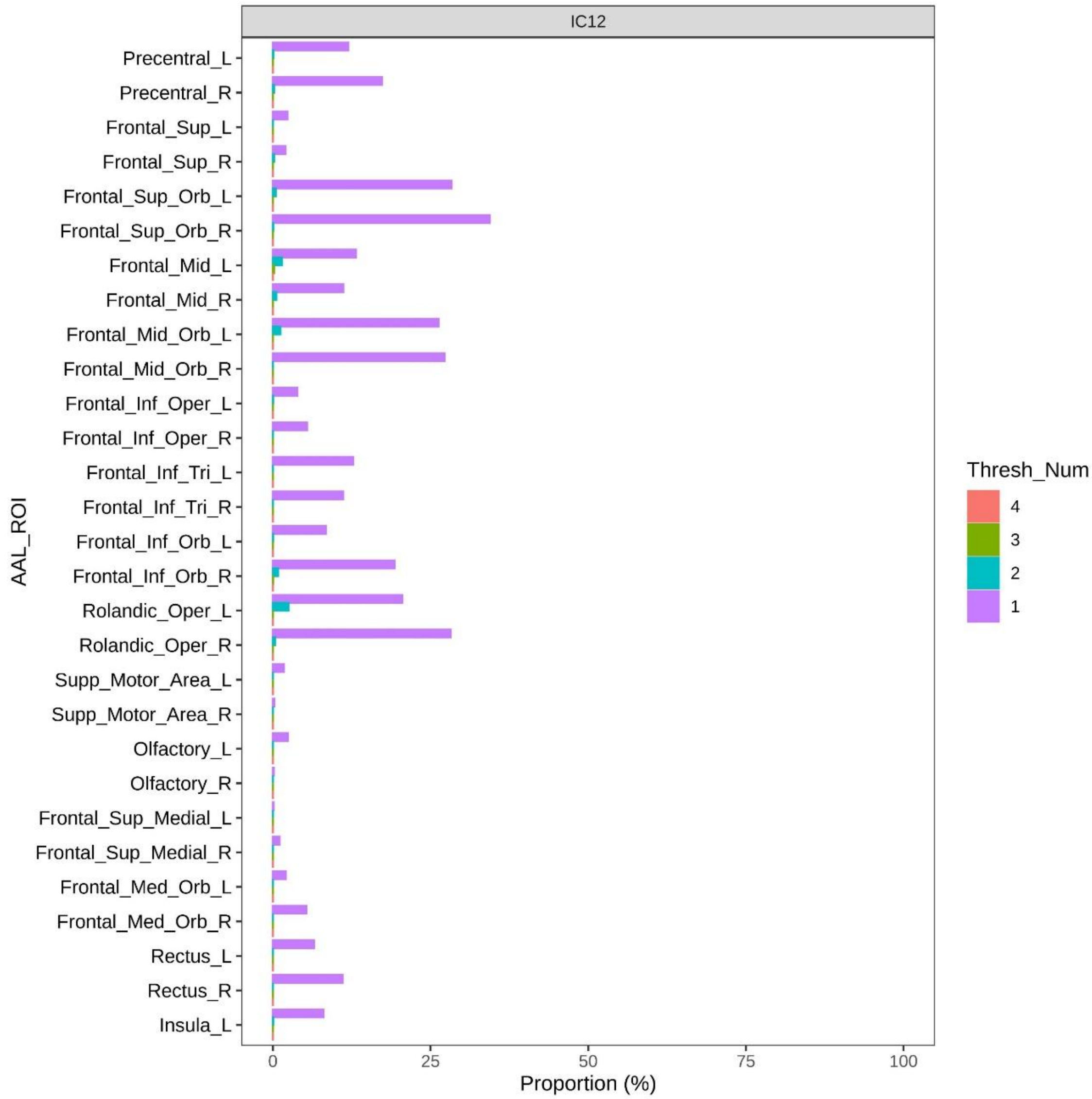

Note. Here, we list the first 29 ROIs included within the AAL3 atlas, determining whether or not these brain regions were included within SBM Component 12. The Y-axis lists the specific AAL3 ROIs and the X-axis displays the proportion of each AAL3 ROI included within SBM Component 6. Results displayed using a Z-threshold of 1.0 (purple), 2.0 (blue), 3.0 (green), and 4.0 (orange).

Supplemental Figure 6

AAL3 ROIs Included Within SBM Component 12

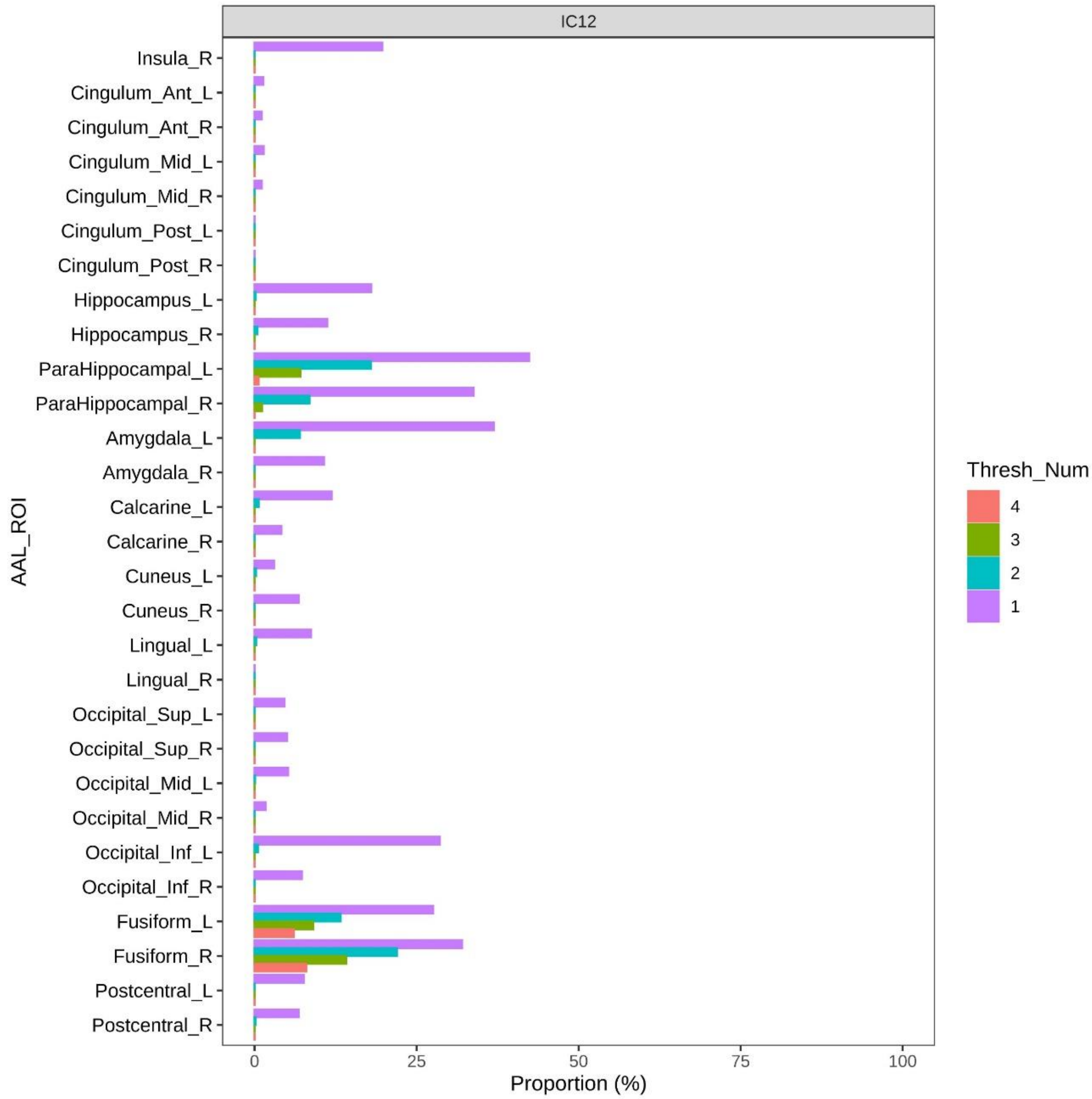

Note. Here, we list the next 29 ROIs included within the AAL3 atlas, determining whether or not these brain regions were included within SBM Component 12. The Y-axis lists the specific AAL3 ROIs and the X-axis displays the proportion of each AAL3 ROI included within SBM Component 6. Results displayed using a Z-threshold of 1.0 (purple), 2.0 (blue), 3.0 (green), and 4.0 (orange).

Supplemental Figure 7

AAL3 ROIs Included Within SBM Component 12

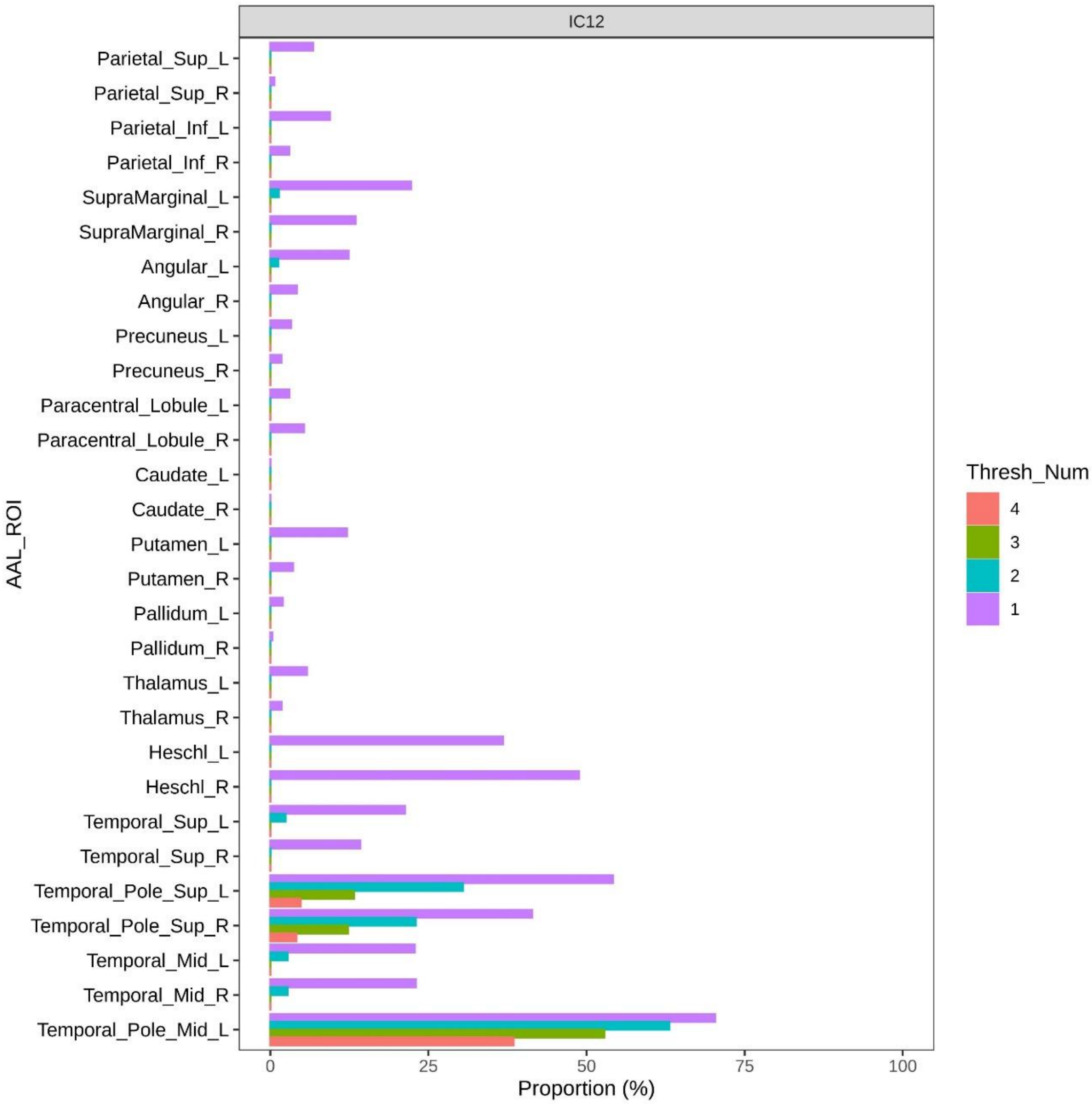

Note. Here, we list the next 29 ROIs included within the AAL3 atlas, determining whether or not these brain regions were included within SBM Component 12. The Y-axis lists the specific AAL3 ROIs and the X-axis displays the proportion of each AAL3 ROI included within SBM Component 6. Results displayed using a Z-threshold of 1.0 (purple), 2.0 (blue), 3.0 (green), and 4.0 (orange).

Supplemental Figure 8

AAL3 ROIs Included Within SBM Component 12

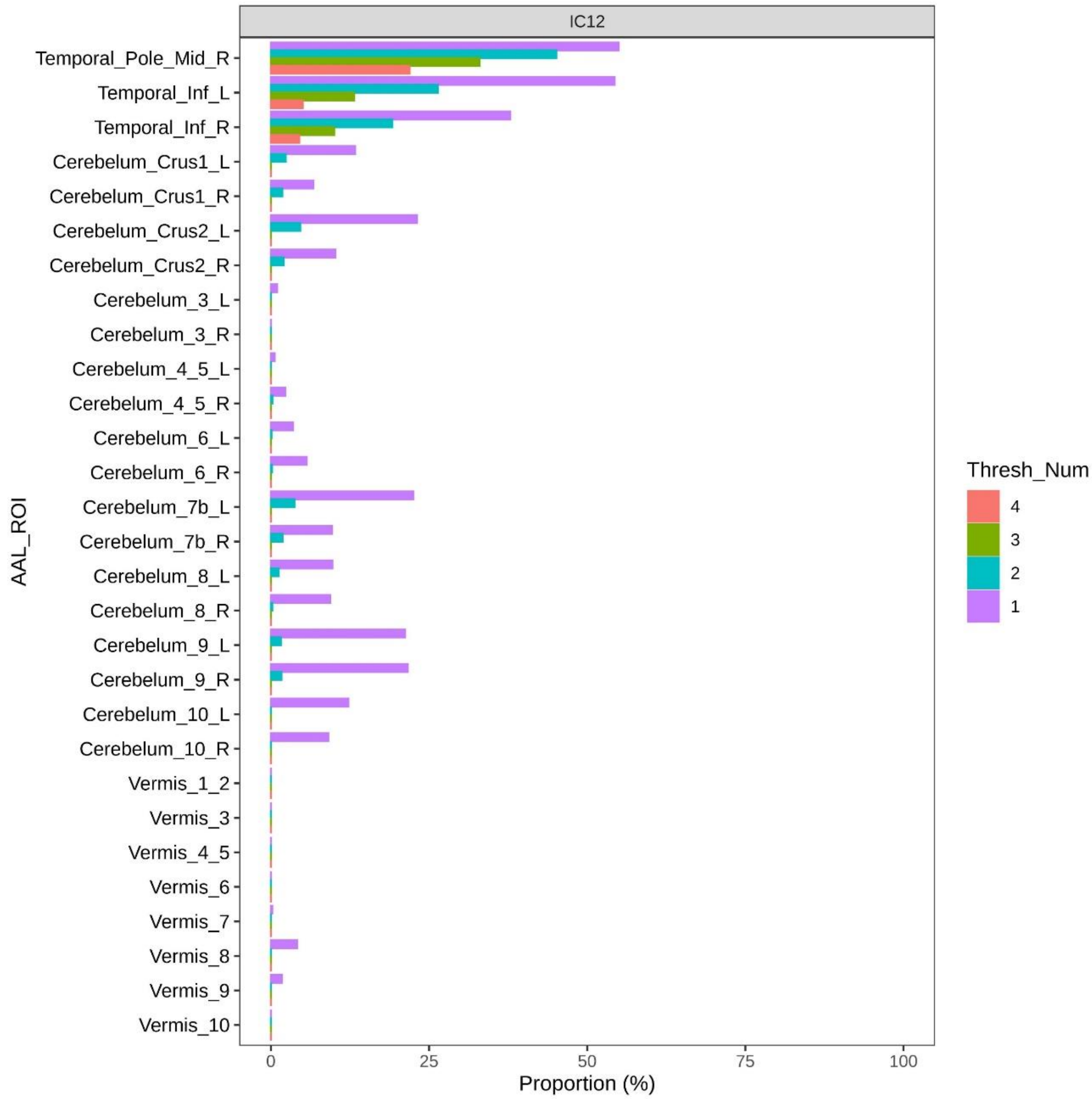

Note. Here, we list the final 29 ROIs included within the AAL3 atlas, determining whether or not these brain regions were included within SBM Component 12. The Y-axis lists the specific AAL3 ROIs and the X-axis displays the proportion of each AAL3 ROI included within SBM Component 6. Results displayed using a Z-threshold of 1.0 (purple), 2.0 (blue), 3.0 (green), and 4.0 (orange).

Supplemental Figure 9

AAL3 ROIs Included Within SBM Component 28

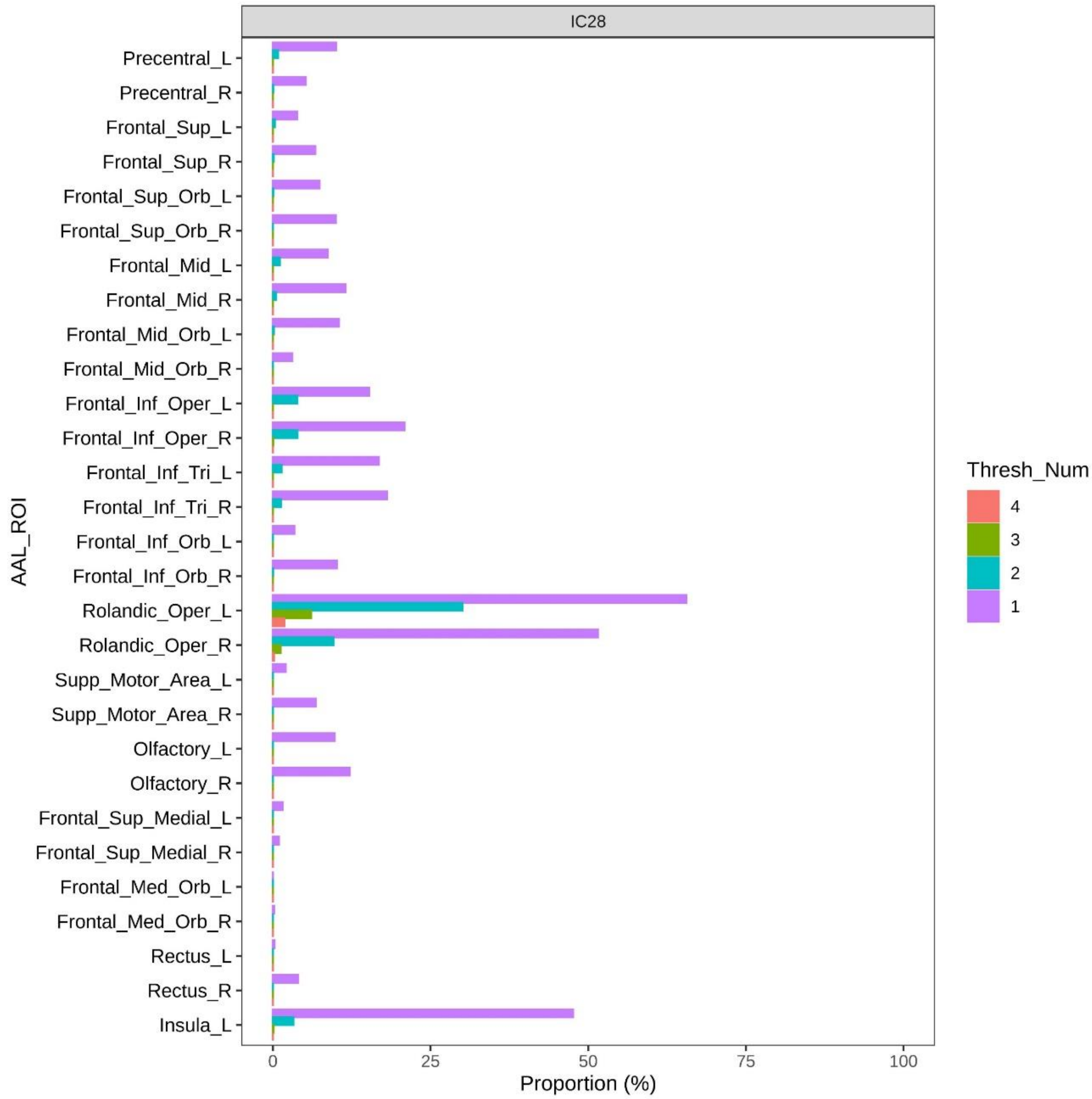

Note. Here, we list the first 29 ROIs included within the AAL3 atlas, determining whether or not these brain regions were included within SBM Component 28. The Y-axis lists the specific AAL3 ROIs and the X-axis displays the proportion of each AAL3 ROI included within SBM Component 6. Results displayed using a Z-threshold of 1.0 (purple), 2.0 (blue), 3.0 (green), and 4.0 (orange).

Supplemental Figure 10

AAL3 ROIs Included Within SBM Component 28

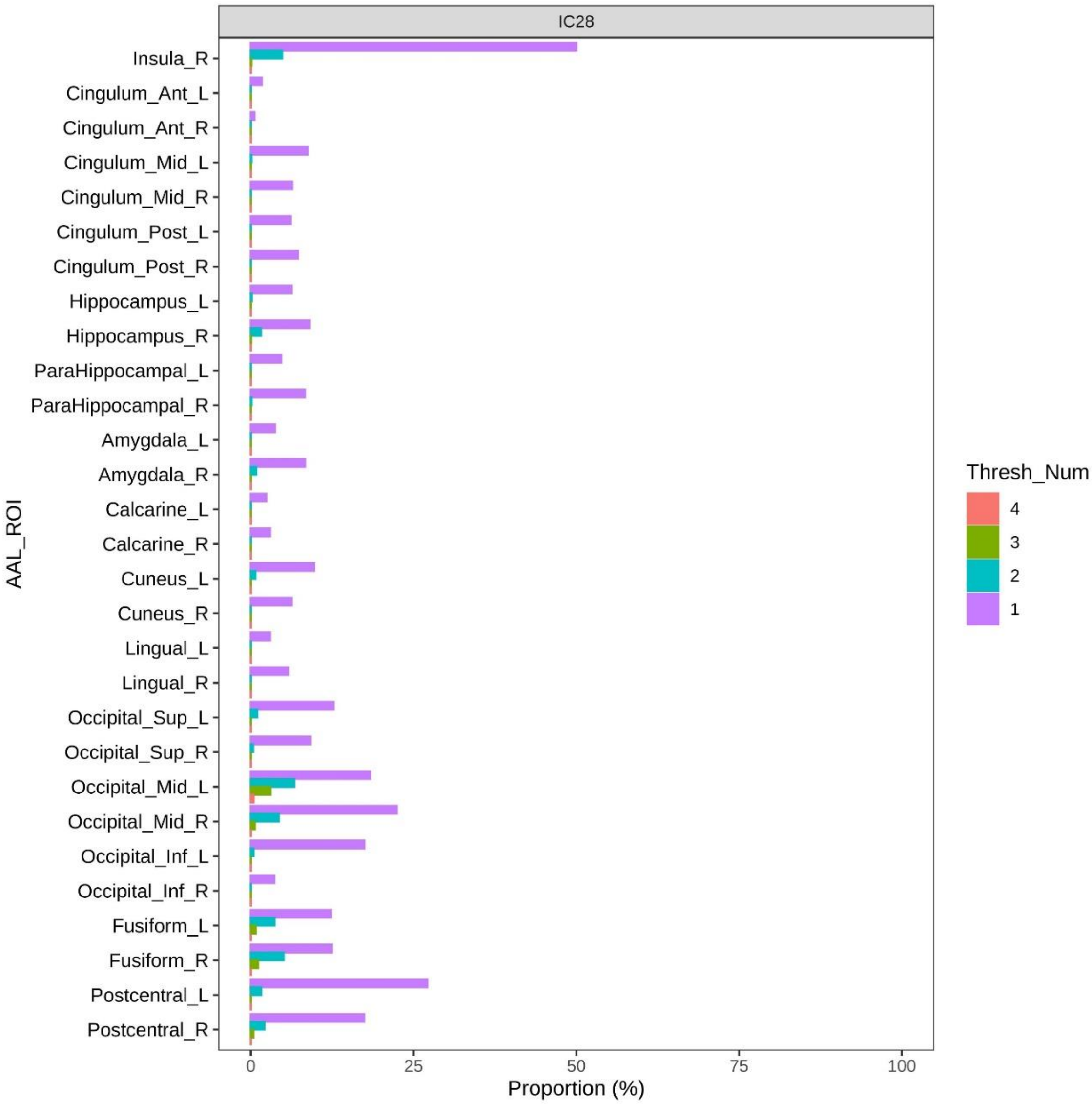

Note. Here, we list the next 29 ROIs included within the AAL3 atlas, determining whether or not these brain regions were included within SBM Component 28. The Y-axis lists the specific AAL3 ROIs and the X-axis displays the proportion of each AAL3 ROI included within SBM Component 6. Results displayed using a Z-threshold of 1.0 (purple), 2.0 (blue), 3.0 (green), and 4.0 (orange).

Supplemental Figure 11

AAL3 ROIs Included Within SBM Component 28

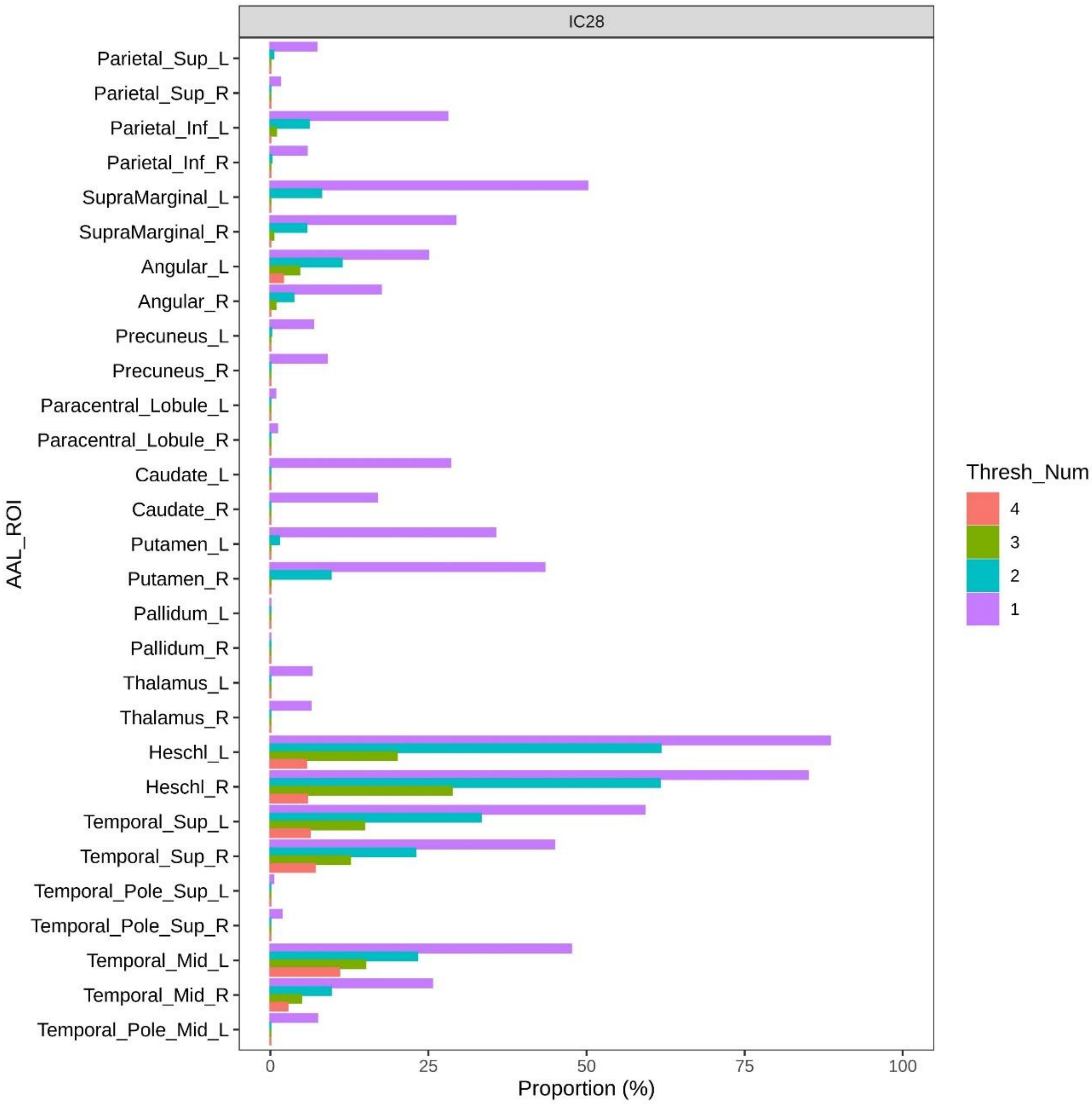

Note. Here, we list the next 29 ROIs included within the AAL3 atlas, determining whether or not these brain regions were included within SBM Component 28. The Y-axis lists the specific AAL3 ROIs and the X-axis displays the proportion of each AAL3 ROI included within SBM Component 6. Results displayed using a Z-threshold of 1.0 (purple), 2.0 (blue), 3.0 (green), and 4.0 (orange).

Supplemental Figure 12

AAL3 ROIs Included Within SBM Component 28

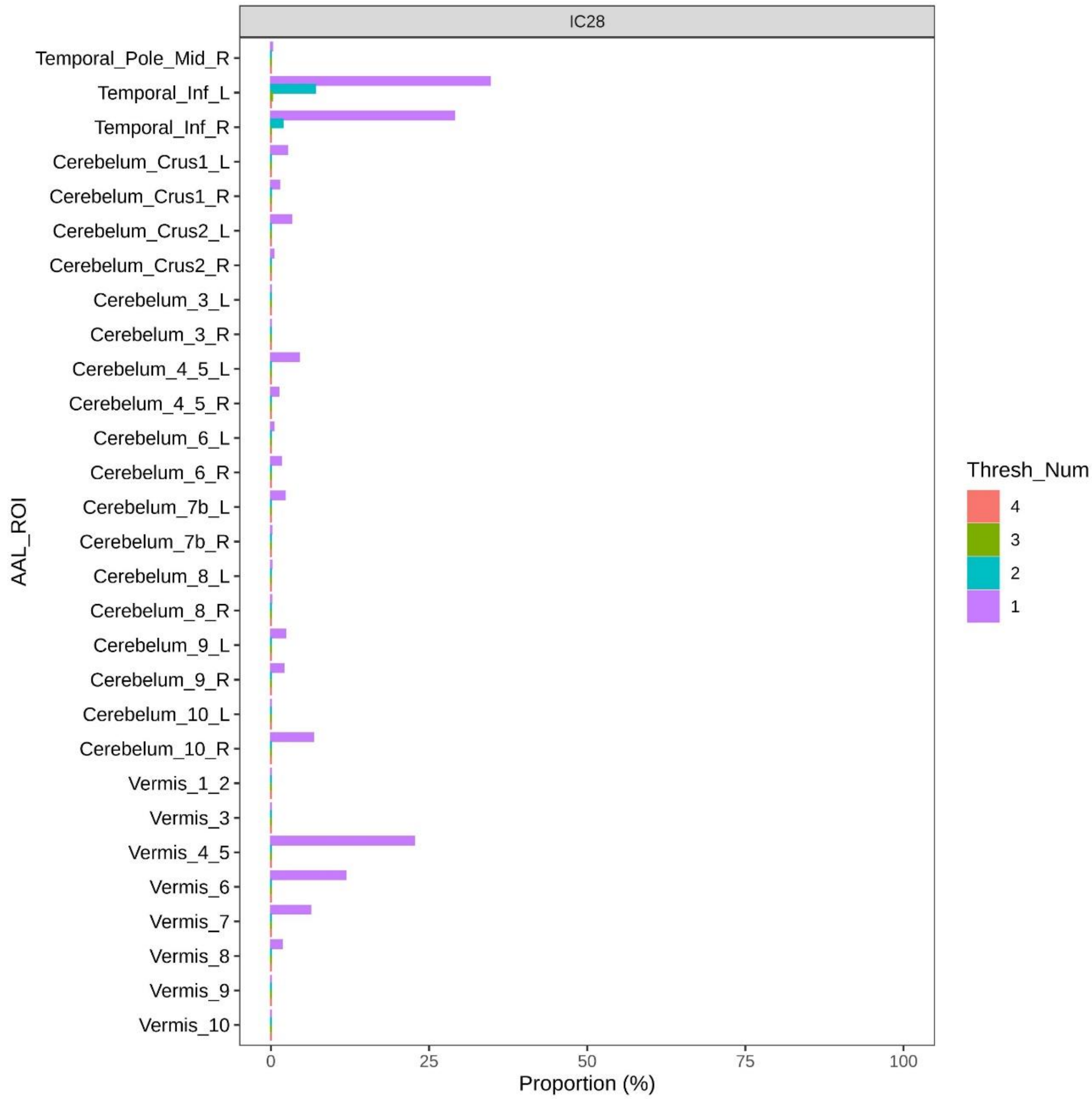

Note. Here, we list the final 29 ROIs included within the AAL3 atlas, determining whether or not these brain regions were included within SBM Component 28. The Y-axis lists the specific AAL3 ROIs and the X-axis displays the proportion of each AAL3 ROI included within SBM Component 6. Results displayed using a Z-threshold of 1.0 (purple), 2.0 (blue), 3.0 (green), and 4.0 (orange).

Supplemental Figure 13

AAL3 ROIs Included Within SBM Component 29

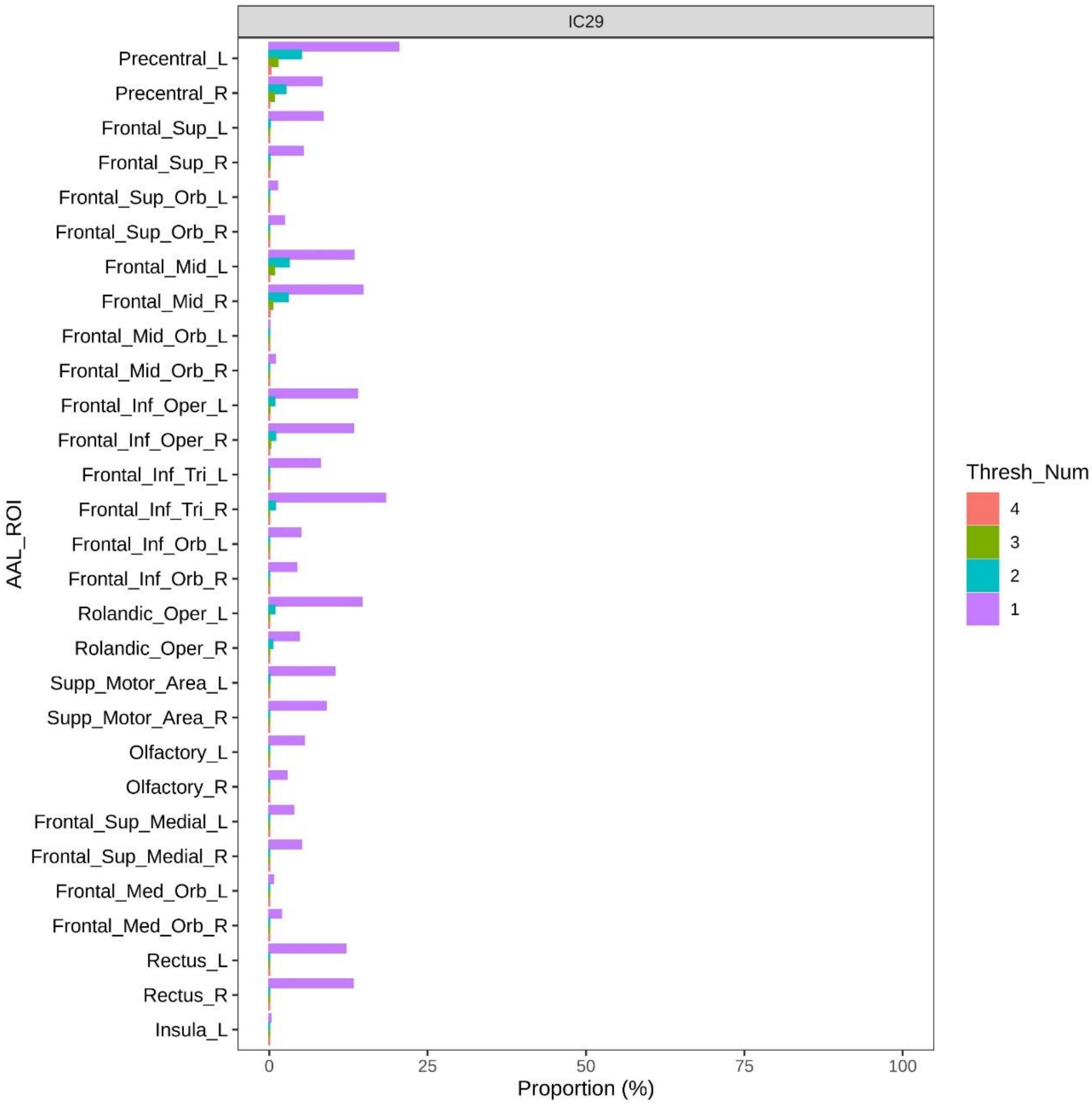

Note. Here, we list the first 29 ROIs included within the AAL3 atlas, determining whether or not these brain regions were included within SBM Component 29. The Y-axis lists the specific AAL3 ROIs and the X-axis displays the proportion of each AAL3 ROI included within SBM Component 6. Results displayed using a Z-threshold of 1.0 (purple), 2.0 (blue), 3.0 (green), and 4.0 (orange).

Supplemental Figure 14

AAL3 ROIs Included Within SBM Component 29

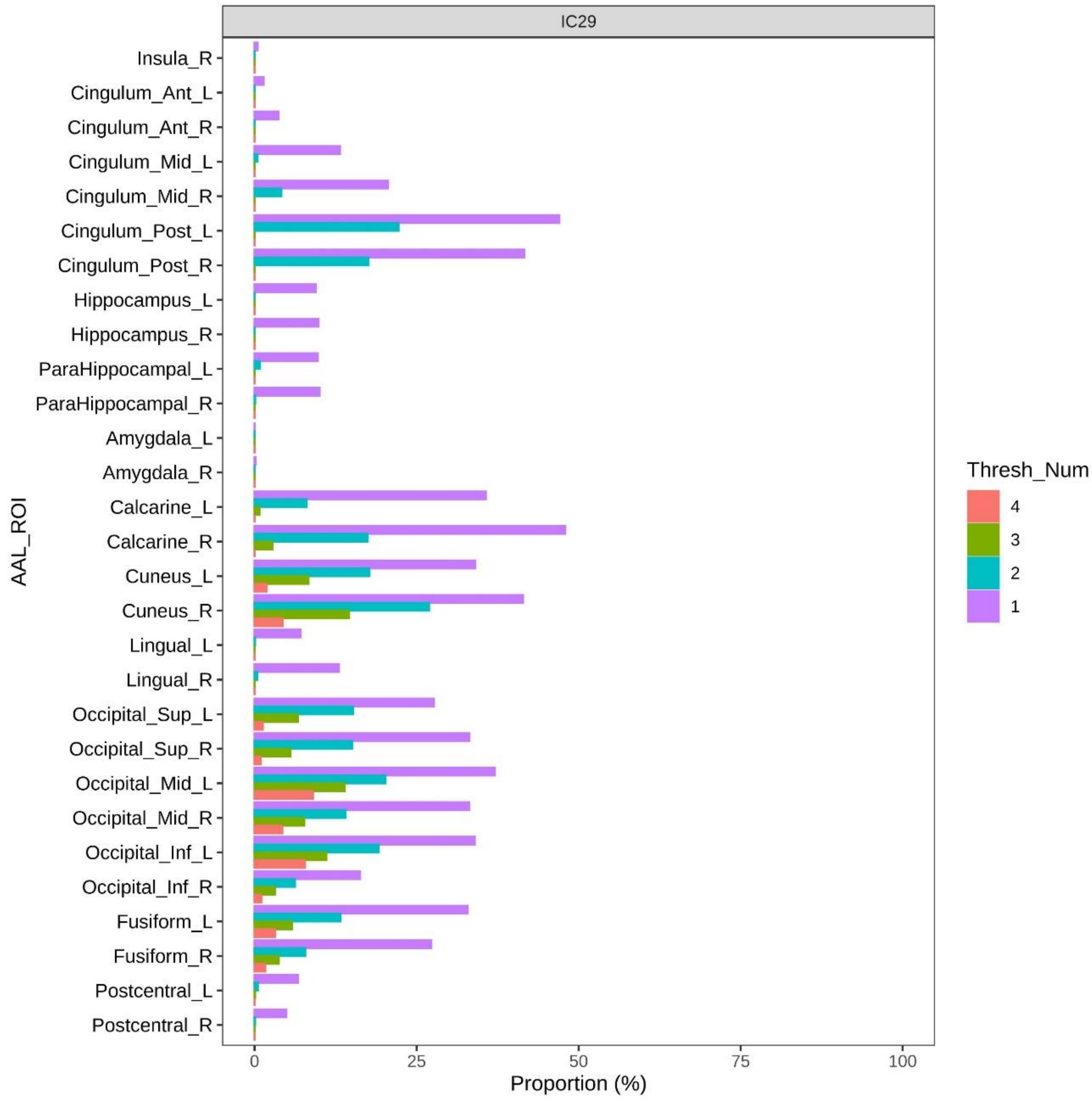

Note. Here, we list the next 29 ROIs included within the AAL3 atlas, determining whether or not these brain regions were included within SBM Component 29. The Y-axis lists the specific AAL3 ROIs and the X-axis displays the proportion of each AAL3 ROI included within SBM Component 6. Results displayed using a Z-threshold of 1.0 (purple), 2.0 (blue), 3.0 (green), and 4.0 (orange).

Supplemental Figure 15

AAL3 ROIs Included Within SBM Component 29

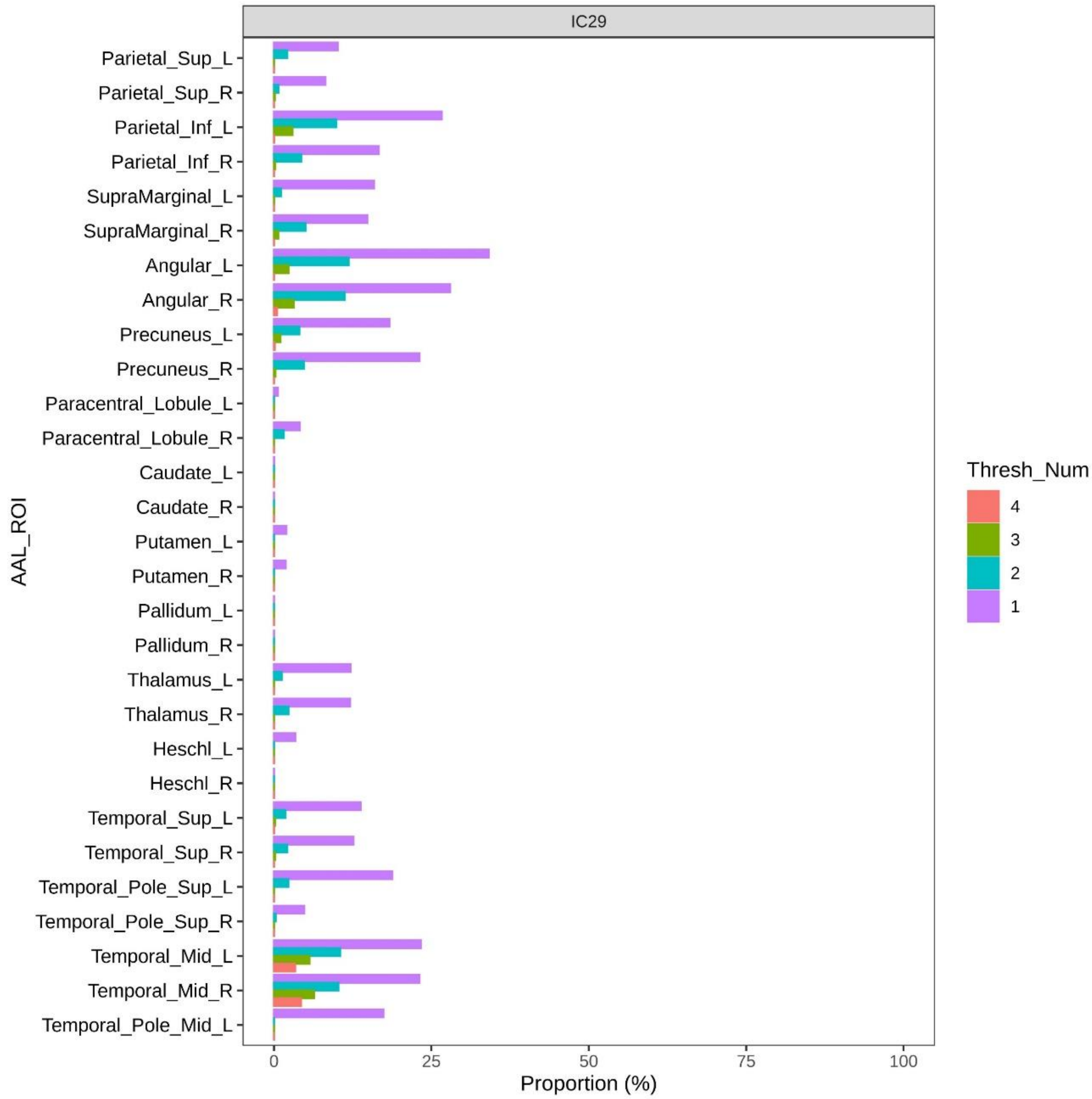

Note. Here, we list the next 29 ROIs included within the AAL3 atlas, determining whether or not these brain regions were included within SBM Component 29. The Y-axis lists the specific AAL3 ROIs and the X-axis displays the proportion of each AAL3 ROI included within SBM Component 6. Results displayed using a Z-threshold of 1.0 (purple), 2.0 (blue), 3.0 (green), and 4.0 (orange).

Supplemental Figure 16

AAL3 ROIs Included Within SBM Component 29

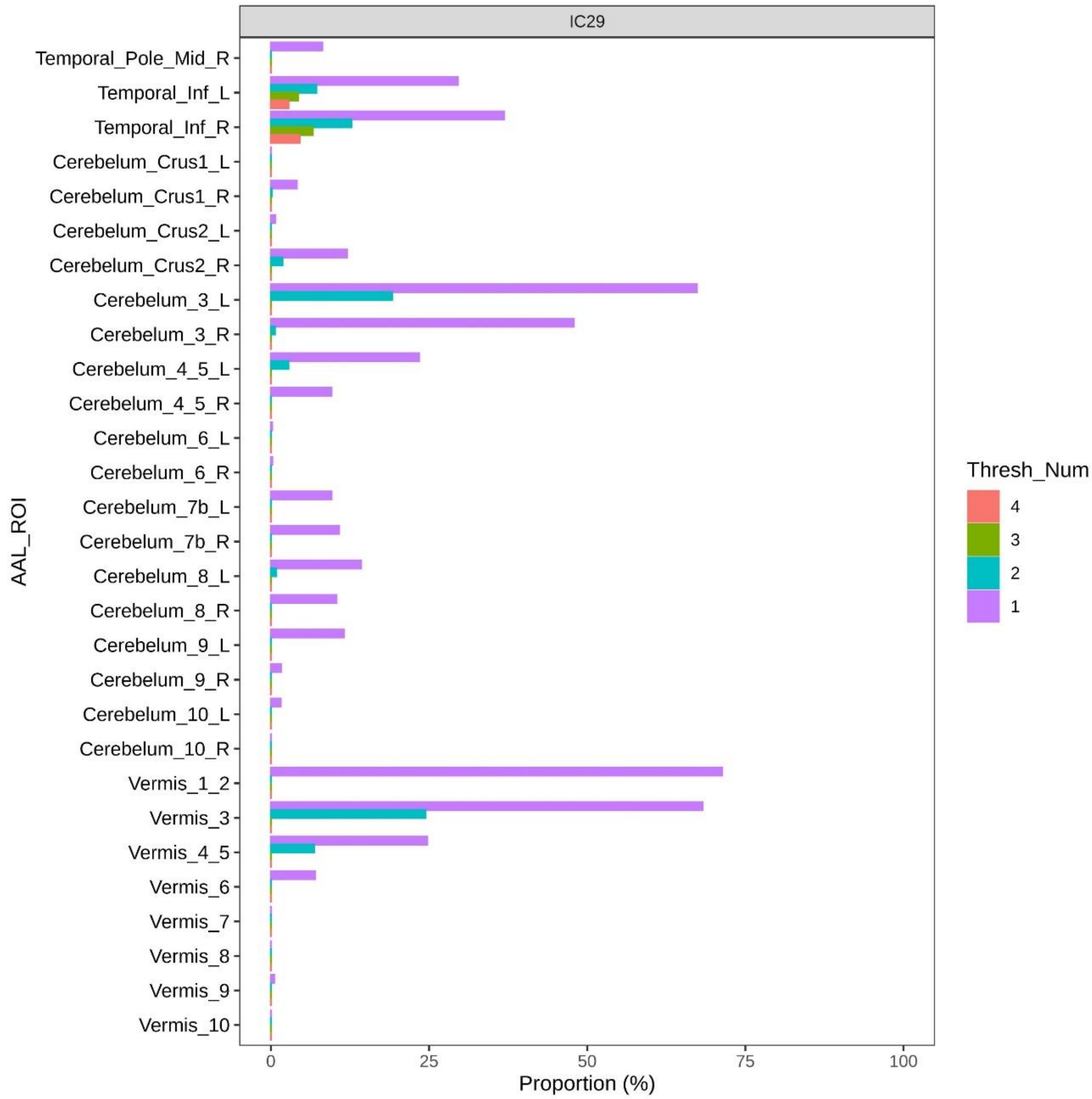

Note. Here, we list the final 29 ROIs included within the AAL3 atlas, determining whether or not these brain regions were included within SBM Component 29. The Y-axis lists the specific AAL3 ROIs and the X-axis displays the proportion of each AAL3 ROI included within SBM Component 6. Results displayed using a Z-threshold of 1.0 (purple), 2.0 (blue), 3.0 (green), and 4.0 (orange).
